# Inferring the diurnal variability of OH radical concentrations over the Amazon from BVOC measurements

**DOI:** 10.1038/s41598-023-41748-4

**Published:** 2023-09-09

**Authors:** A. Ringsdorf, A. Edtbauer, J. Vilà-Guerau de Arellano, E. Y. Pfannerstill, S. Gromov, V. Kumar, A. Pozzer, S. Wolff, A. Tsokankunku, M. Soergel, M. O. Sá, A. Araújo, F. Ditas, C. Poehlker, J. Lelieveld, J. Williams

**Affiliations:** 1 Department of Atmospheric Chemistry, Max Planck Institute for Chemistry, Mainz, Germany; 2 Meteorology and Air Quality Section, Wageningen University, Wageningen, The Netherlands; 3 Satellite Remote Sensing Group, Max Planck Institute for Chemistry, Mainz, Germany; 4 Instituto Nacional de Pesquisas da Amazônia (INPA), Manaus, CEP 69067-375 Brazil; 5 Empresa Brasileira de Pesquisa Agropecuária (Embrapa) Amazonia Oriental, Belém, CEP 66095-100 Brazil; 6 Department of Multiphase Chemistry, Max Planck Institute for Chemistry, Mainz, Germany; 7 Climate and Atmosphere Research Center, The Cyprus Institute, 1645 Nicosia, Cyprus; 8 Present Address: Friedrich-Alexander-Universität Erlangen-Nürnberg, Sachgebiet Arbeitssicherheit, Erlangen, Germany; 9 Present Address: Hessian Agency for Nature Conservation, Environment and Geology, Wiesbaden, Germany

**Keywords:** Environmental chemistry, Climate sciences, Atmospheric science, Atmospheric chemistry, Atmospheric dynamics

## Abstract

The atmospheric oxidation of biogenic volatile organic compounds (BVOC) by OH radicals over tropical rainforests impacts local particle production and the lifetime of globally distributed chemically and radiatively active gases. For the pristine Amazon rainforest during the dry season, we empirically determined the diurnal OH radical variability at the forest-atmosphere interface region between 80 and 325 m from 07:00 to 15:00 LT using BVOC measurements. A dynamic time warping approach was applied showing that median averaged mixing times between 80 to 325 m decrease from 105 to 15 min over this time period. The inferred OH concentrations show evidence for an early morning OH peak (07:00–08:00 LT) and an OH maximum (14:00 LT) reaching 2.2 (0.2, 3.8) × 10^6 ^molecules cm^−3^ controlled by the coupling between BVOC emission fluxes, nocturnal NO_x_ accumulation, convective turbulence, air chemistry and photolysis rates. The results were evaluated with a turbulence resolving transport (DALES), a regional scale (WRF-Chem) and a global (EMAC) atmospheric chemistry model.

## Introduction

The hydroxyl radical (OH) is the main atmospheric oxidant for trace gases in the global atmosphere^1^. By initializing the removal of natural and anthropogenic hazardous and radiatively active gases (e.g. CH_4_) it is an important factor for human health and global climate^2^. The primary atmospheric source of OH is a pathway of ozone photolysis which forms O(^1^D) atoms, that can react rapidly with water to produce two OH radicals^3^. As a result most OH initiated atmospheric oxidation occurs in the tropical lower troposphere where high insolation and water vapor combine with ubiquitous ozone to generate high OH production rates^1^. However, in the same regions, tropical forests provide the largest global emission source of biogenic volatile organic compounds (BVOC) which react readily with OH^4,5^. Therefore, a critical interface region exists directly above tropical forests where strong sources and sinks of OH are co-located, the net result being key to the lifetime of many gases^1,6^. Due to their coarse resolution (typical grid cell sizes > 25 km with about 10 horizontal layers in the atmospheric boundary layer (ABL)), global atmospheric models are known to experience problems in this region as the strong VOC emissions suppress OH and cause overestimates of BVOC mixing ratios relative to measurements^7^–^11^.

Simultaneous airborne measurements of OH, hydroperoxyl radical (HO_2_) and BVOC over a tropical rainforest in 2005 led to the closer scrutiny of isoprene oxidation chemistry, for additional OH sources and recycling routes ^6^. Although the OH measurements reported were subsequently found to be artificially high due to an instrumental interference^12^, theoretical studies^13^, mechanistic and modelling investigations^6,14^, laboratory chamber-based oxidation studies^15^ and kinetic experiments^16^ revealed underestimated or even overlooked routes through which OH was recycled during the oxidation of isoprene.

**Figure 1:**
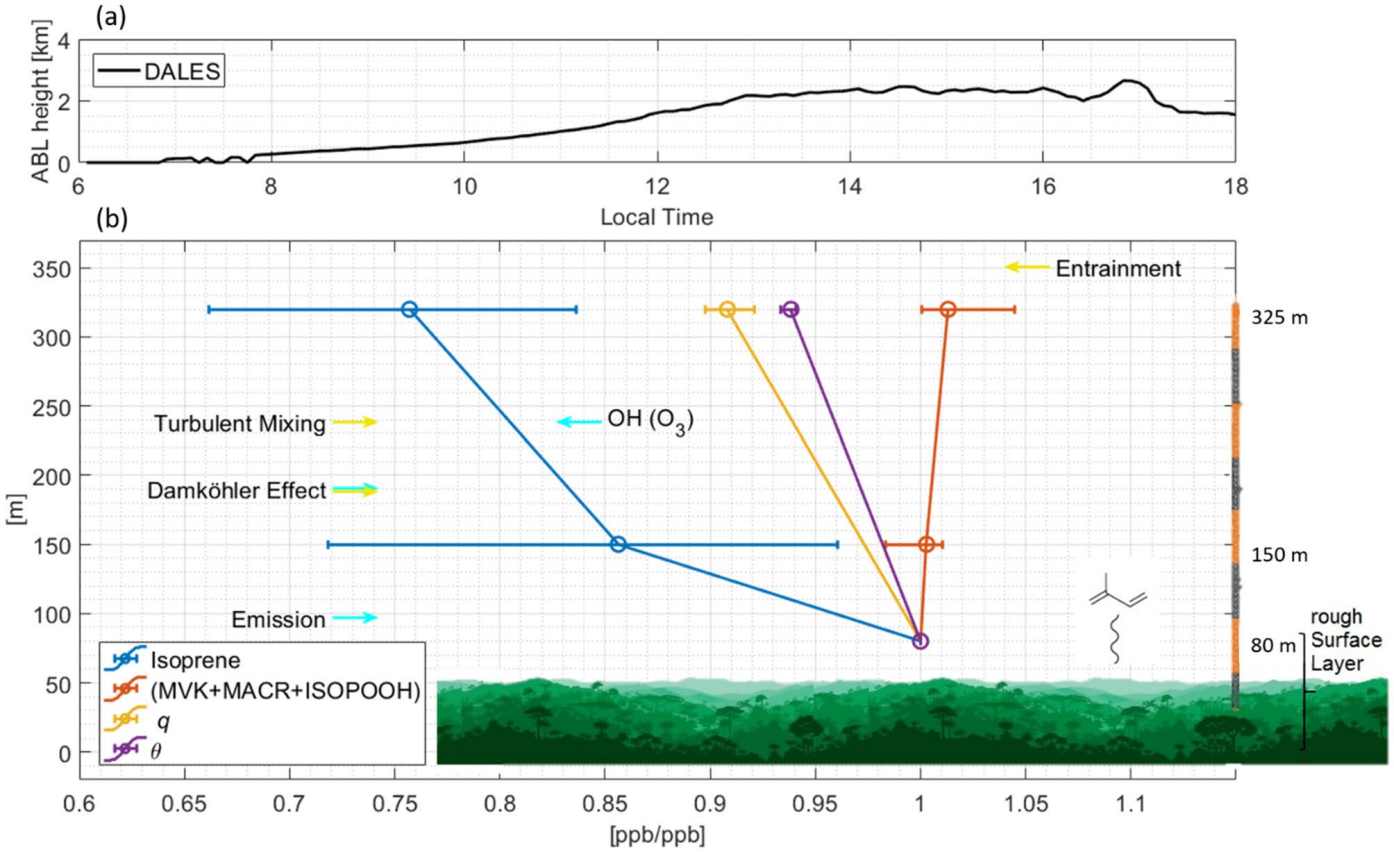
Overview of the processes that govern the vertical distribution of variables observed at ATTO (**a**) Time series of the boundary layer height simulated by DALES (**b**) Schematic sketch of the processes governing the vertical gradient of reactive trace gases, here isoprene, emitted from the Amazon forest. Dynamic processes are marked with yellow arrows while blue arrows indicate chemistry related processes. The Damköhler effect has two arrows since it quantifies the relation between the turbulent and chemistry time scales. The relative vertical gradients of isoprene, the isoprene oxidation products Methyl Vinyl Ketone + Methacrolein + Isoprene Hydroxyhydroperoxides (ISOPOOH), specific humidity *q* and potential temperature *θ* observed at ATTO are median averages at 12:00–14:00. The error bars indicate the (0.15, 0.85) quantiles.

In this paper we present a new empirical derivation of the OH abundance and diurnal variation above a largely pristine part of the Amazonian rainforest^17^ characterized by low nitrogen oxides (NO_x_) conditions (NO < 150 ppt at 80 m). To infer an OH radical abundance, other than by direct measurement, requires measurement of a change in concentration of one or more BVOCs with a known OH reaction rate coefficient, and to accurately know the reaction time (period during which reactive molecules are exposed to OH oxidation). Here a change of the BVOC concentration with height above a rainforest is studied. We have measured BVOC concentrations on a tall tower at two heights (80 and 325 m) and investigate to which extent the concentration gradients are influenced by oxidation chemistry, turbulent mixing, emission and dilution due to transport from above (Fig. 1). Several indirect estimates of OH have been made in the past using BVOC concentrations and fluxes acquired from towers and aircraft in conjunction with models^18,19^. However, all have approximated the reaction time in the vertical direction through use of the convective velocity (${w}_{*}$) of the ABL. In order to better ascertain the timescale, in this study we approach the vertical reaction time by applying the Dynamic Time Warping (DTW) analysis alignment method^20^ to simultaneously observed meteorological data (see method for details).

**Figure 2:**
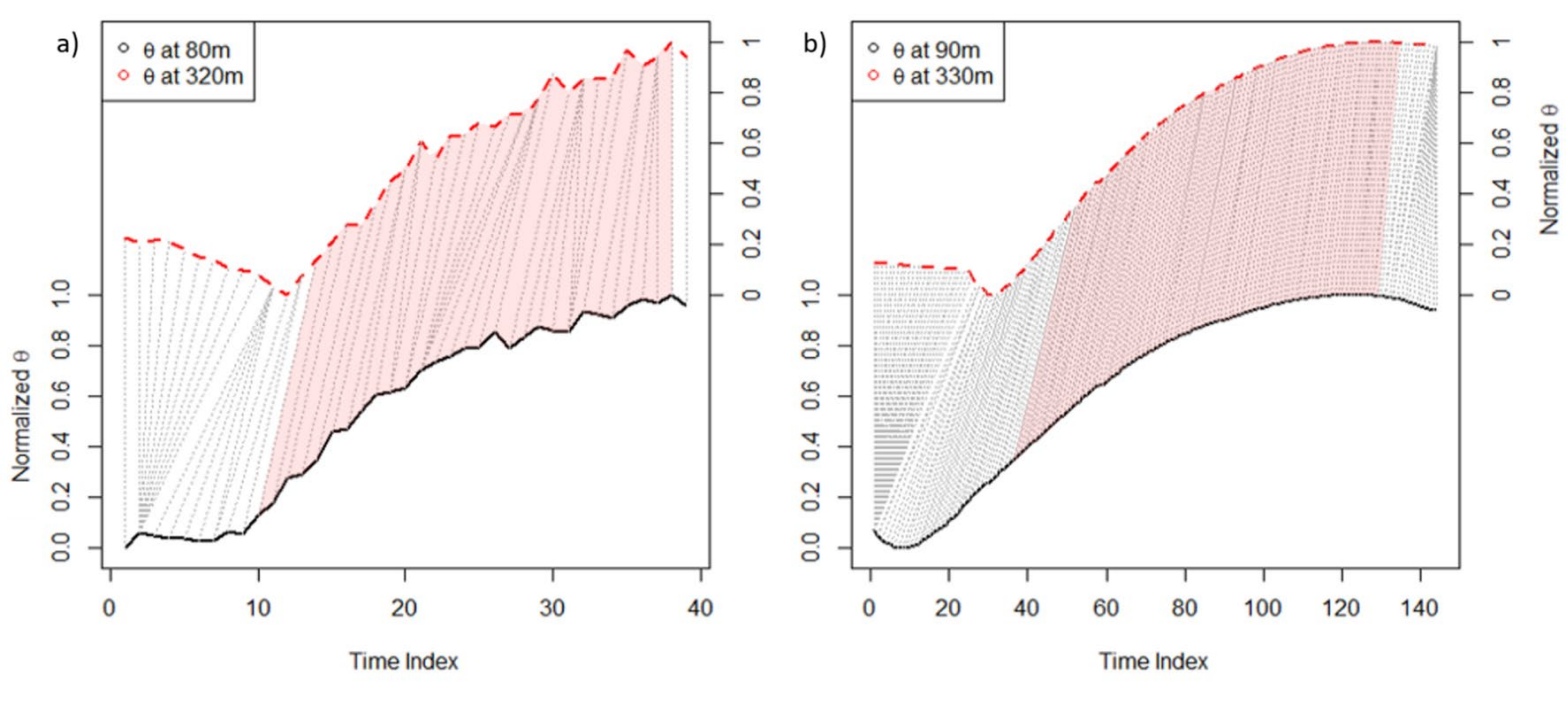
Example of Dynamical Time Warping alignment of the normalized time series of potential temperature *θ* between zero and one over the course of one day. The dotted lines visualize their alignment pointwise. Convective ABL conditions apply only for the marked alignments, thus the mixing timescale is derived only from this period. This precludes edge effects. The index represents the time steps depending on their resolution. (**a**) *θ* observed at the measurement heights. The period under analysis includes 23 dry season days. (**b**) *θ* at two corresponding levels simulated for a representative day with DALES.

‘Dynamical Time Warping’ (DTW) in conjunction with a chemically inert tracer like the potential temperature (*θ*), which varies with time, allows quantification of the time offset between the timeseries at two reference heights (Fig. 2) under diurnal turbulent convective conditions. Air heated at the top of the canopy by conduction is transported vertically via turbulent convection, creating turbulent buoyant plumes that are organized in warm upward and cold downward motions. At the interfaces of these plumes lateral mixing occurs between the motions, resulting in a warming of the boundary layer. This heating is observed in the time offset of *θ* between 80 and 325 m. After normalizing the observed daily cycles of *θ* according to their magnitude, the time offset is extracted by DTW as it projects one timeseries onto another (using a cost minimization matrix). Thus, the DTW method provides a mixing timescale that approaches the time that air is exposed to oxidation. Mixing of airmasses containing isoprene and OH also result from the ensemble of the upward and downward turbulent motions. We exploit the daily time differences observed in *θ* at each height under diurnal convective conditions averaged over 23 days to extract a statistically robust, continuous and representative vertical mixing time as a function of time-of-day. Combining the extracted time with isoprene concentrations at 80 and 325 m and the corresponding formation of its oxidation products (IsoO, here: Methyl Vinyl Ketone (MVK) + Methacrolein (MACR) + Isoprene Hydroxyhydroperoxides (ISOPOOH)) allows calculation of the OH concentration between 80 and 325 m and from 07:00–15:00 LT with two different methods. The empirically derived OH concentrations were then evaluated against those simulated by three different atmospheric chemistry models: the turbulence resolving Dutch Atmospheric Large-Eddy Simulation (DALES)^21^ the regional scale forecasting model WRF-Chem^22^ and the atmospheric chemistry general circulation model EMAC^23,24^. Since DALES is able to calculate the most energetic turbulent eddies in the roughness sublayer, we use it to independently test the uncertainties of the empirical methods using control numerical experiments.

OH concentrations are derived between 80 and 325 m in the ABL, which extends from just above the canopy at about 37 m^18^ to about 2 km between sunrise at 06:00 and 14:00 local time (UTC—4 h) (Fig. 1a). During the night the lower atmosphere is stably stratified with positive vertical gradients of *θ*. After sunrise, the positive sensible and latent heat fluxes lead to the formation of the convective boundary layer via thermal expansion of air that triggers buoyancy, i.e. density instabilities that lead to convective turbulence. Turbulent eddies that form coherent structures characterized by upward thermal and downward subsidence motions are induced^25,26^. During the transport and mixing chemical reactions take place^27^. Strong insulation and rising surface temperature throughout the day lead to increasing buoyancy and turbulence until the afternoon transition^26^. The warming and growth of the ABL have implications for the derived mixing timescale. When considering the free convective scaling velocity of single updrafts, w*, an increase with increasing buoyancy is observed^25^. Direct comparison between w* and the mixing timescale derived here is however not possible. The DTW-derived timescale considers the heating of the boundary layer, which represents the ensemble of thermal upwards and subsiding transport. Further, the measurements taken at around 80 m are likely to be inside the roughness-sublayer that spans within the ABL from the ground to 2–3 times the canopy height (37 m)^28,29^. Turbulence there differs from the layers aloft with strongly inhomogeneous flows of sweeping and ejection motions induced by deceleration of wind at the rough canopy surface^30,31^. The timescale of w* represents that of individual large eddies in reaching the top of the ABL.

## Results and discussion

The BVOC measurements reported here were taken at 80, 150 and 325 m from the ATTO tower sited amidst the pristine Amazon rainforest. Isoprene and its combined oxidation products IsoO were measured using PTR-ToF–MS at m/z 69.069 and 71.049. In order to investigate the vertical evolution of BVOC above the forest canopy, air from 80, 150 and 325 m was measured sequentially for five minutes at each height generating four datapoints per hour at each level. Isoprene was chosen for the OH radical concentration determination as it is an abundant, primary emitted BVOC without secondary sources and predominantly oxidized by OH^32^. Meteorological variables were observed on the ATTO tower at 321 m and the 80 m tall walk-up tower on the same site at 81 m. BVOC and meteorological variables were measured continuously but the method to infer OH concentrations is restricted to convective periods which are defined by *θ*_325m_ < *θ*_80m_. This condition ensures a net-upward propagation of thermal energy that can be converted into a mixing time. The maximum of *θ* at 80 m serves as an upper limit after noon. By then the declining insolation and concomitant decreasing buoyancy force leads to stabilization rendering the method inoperable. The vertical distribution of BVOC at ATTO is affected by the complex dynamic regime at the atmosphere-canopy interface^29^ as well as by chemical processing. Measured dry season vertical mixing ratio profiles of isoprene, IsoO, specific humidity (*q*) and *θ* are shown at noon in Fig. 1b. Isoprene, *q* and *θ* show the largest values at the canopy level, where their source is located, decreasing with altitude due to multiple processes, including emission strength changes, mixing and chemical reactions. The highly reactive isoprene decreases by 14.4% from 80 to 150 m and 24.3% up to 325 m. Conversely IsoO, the OH photochemical product slightly increases with height in the measurements, due to the interplay of isoprene oxidation and turbulent mixing, that implies up- and downwards movement and mixing of more or less aged emissions. Observations reported by Rinne et al.^33^ indicated little chemical degradation of isoprene above the boreal forest up to 22 m, however, OH production rates there and turbulence intensity are expected to be substantially lower than in the tropics. Rinne et al.^33^ also noted that highly reactive sesquiterpene emissions from a boreal forest would be chemically oxidized faster than they can be transported aloft^33^. This is also in agreement with this study as although sesquiterpenes have been measured previously at ground level at the ATTO site^34,35^, these species were not quantifiable at 325 m. The sensitivity of the vertical isoprene gradient to OH oxidation chemistry and mixing is investigated with the use of the turbulent-resolved simulation technique DALES later in this study.

The vertical concentration gradient of isoprene, observed over 80–325 m becomes steeper with height (i.e. large concentration differences between top and bottom) due to three processes: surface emission, entrainment of isoprene-free air from above and OH oxidation^36,37^. In contrast turbulent mixing causes two effects that both act to weaken the vertical gradient. Firstly, the establishment of the concentration gradient is counteracted by the mixing of isoprene rich upward motions with isoprene poor air transported from the downward subsident motions. This asymmetry in the transport also leads to segregation due to the inhomogeneous mixing^37,38^. The segregation of isoprene lowers the effective reaction rate ($k_{{{\text{iso}} + {\text{OH}}}} )$, and as a result, the impact of OH oxidation on the vertical gradient in isoprene is weakened^38,39^.

In order to quantify the competition and relative dominance of dynamical processes and chemical processes over the whole day the Damköhler number (Da) is introduced. This term is defined as:

1 $${\text{Da }} = \frac{{\tau_{{\text{D}}} }}{{\tau_{{\text{C}}} }} = \frac{{{\text{mixing time }}\left( {\text{s}} \right)}}{{\left( {\left[ {{\text{OH}}} \right]{ }k_{{{\text{iso}} + {\text{OH}}}} } \right)^{ - 1} { }\left( {\text{s}} \right)}}$$

**Figure 3:**
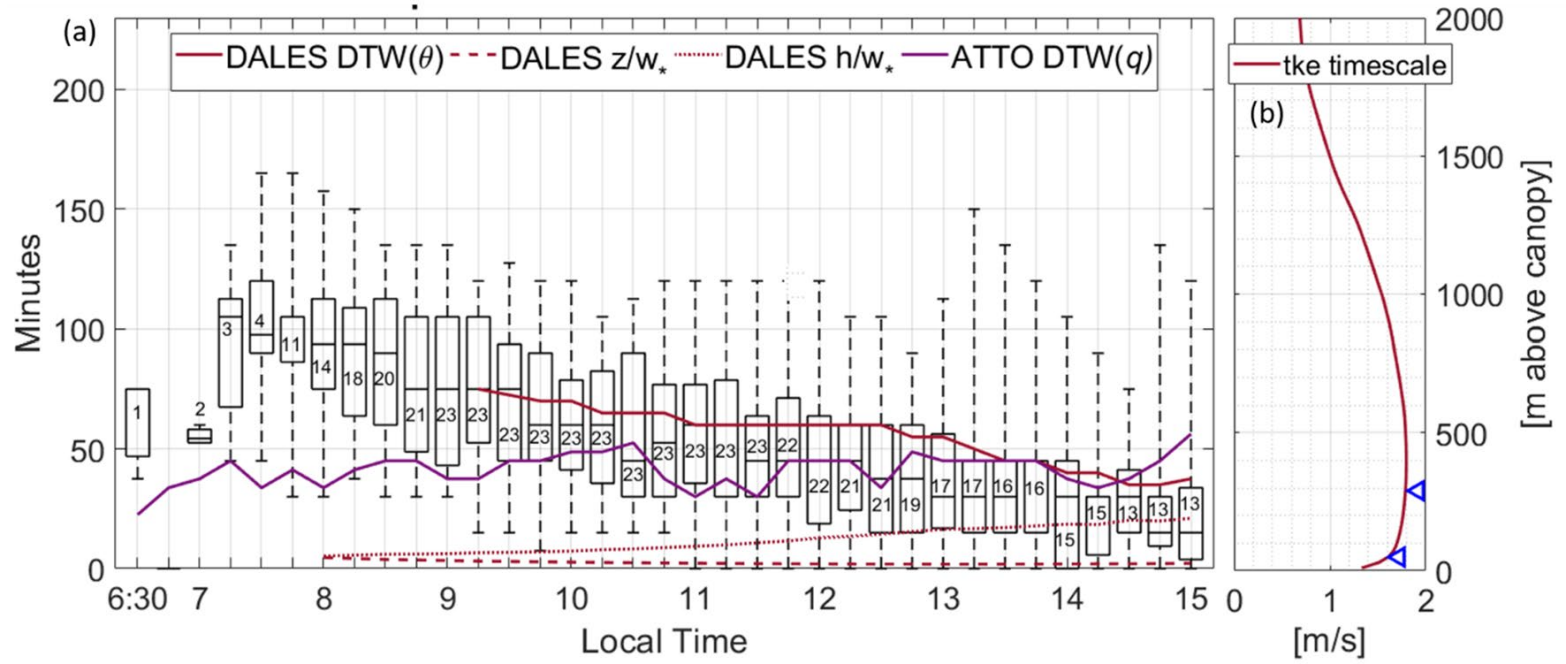
Variations of the mixing time with time of day and height (**a**) The box- whisker plots shows the median and the range of the 75th percentile of the Dynamical Time Warping derived mixing time from ATTO observations. Whiskers include all datapoints. The numbers in the boxes describe the numbers of days used to generate the box and whiskers. The dark red line is the DTW derived mixing time with potential temperature simulated at the corresponding altitude levels of DALES. Timescales calculated from the convective velocity with the ABL height (*h*) and sampling height (*z*) are included as dashed lines. The purple line shows the DTW derived mixing time from observed timeseries of *q*. (**b**) The distribution of the vertical velocity timescale based on turbulent kinetic energy (TKE) by DALES at 12:00 LT. Blue arrows point at the ATTO sampling heights.

It is a dimensionless number that compares the timescale of dynamics $\tau_{{\text{D}}}$ to that of chemistry $\tau_{{\text{C}}}$, with values of unity indicative of a competitive regime and a maximum intensity of segregation^38^. Within equation (Eq. 1) the timescale of dynamics is typically represented by the convection timescale *t*

2 $$t = \frac{h}{{w_{*} }}$$

where *h* is the boundary layer height and $w_{*}$ the convective velocity of single updrafts. As the aim is to investigate the conditions over the vertical extent of the ATTO tower, the DTW derived mixing time is introduced in the following section (Figs. 2 and 3) and used instead. It is shown that ensemble turbulent motions in the atmosphere canopy interface region happen on longer timescales than estimated by *t* which is critically important for the OH estimation.

### Mixing Time in the low ABL

The DTW technique as described within the methods section was applied to the observed time-series of potential temperature *θ* for a total of 23 dry season days from October 2018 and September 2019 between 6:00 and 18:00 LT. This is shown for one example day in Fig. 2 where normalized *θ* values at 80 and 325 m are allocated to find the time index difference corresponding to warming at both heights. The warming is a result of lateral mixing induced by turbulent buoyant plumes.

As precipitation events interfere with the vertical distribution of *θ* a filter is applied to remove such periods (30.8% of data). To infer OH concentrations from the oxidative change of isoprene concentrations with height and DTW derived mixing times requires convective conditions. Periods with a stable stratification (*θ*_325m_ > *θ*_80m_) were therefore excluded. This affects the morning transition from the stable nocturnal ABL to the convectively mixed ABL. The daily maximum of *θ* at 80 m was taken as the later limit for the calculation of OH, which occurred at the latest by 15:00 LT. The time restriction was also supported by the sign of the non-dimensional z/L number (sampling height *z* divided by the Obukhov length *L*) observed at ATTO (Supplementary Fig. S1). It indicates the stability of the ABL with positive values representing a stable stratification^25^. The Obukhov length was estimated from the meteorological flux measurements at 81 m.

Median averaged mixing times from 80 to 325 m ranged from 15 to 105 min (Fig. 3). Large mixing timescales are found in the morning when thermal and shear driven motions are slow due to the sensible and latent heat fluxes being still small. Throughout the day the values of the mixing timescale decrease as turbulent convection becomes more dominant. To further complete the assessment of the DTW method, it was also applied to the observed timeseries of specific humidity *q* at the sampling heights. Despite *θ* being related to the sensible heat flux and therefore to the active part of turbulence, and *q* in contrast being transported by this turbulence, the time scale derived with *q* has similar values. It varies around 45 min and is comprised within the 75^th^ percentile of the timescale derived with *θ* after 10:00 LT. Before that time both timescales show considerable differences, likely because the timeseries of *q* are altered due to condensation and evaporation in low level clouds that are often observed visually in the morning over the rainforest.

The DTW-derived mixing times between the two equivalent *θ* levels of DALES (90–330 m) from 9:00 to 15:00 LT are slightly higher than derived for ATTO but comprised within the day to day variation. DALES is constrained to the isoprene observed at ATTO and explicitly resolves turbulence above the canopy, without resolving explicitly the interactions between the tall canopy and the above layer. As a result, the characteristic motions of ejection and sweeping at the interface are not resolved. The canopy is represented by a bulk layer with a high roughness length to account for changes in turbulence just above the canopy top^40^. Thus, using DALES, the influence of the tall canopy on turbulent transport above cannot be disentangled. Friction velocity however, simulated by DALES agrees well with observations above the Amazon forest^40^.

For comparison the timescale *t* calculated from the convective velocity (Eq. 2) is shown in Fig. 3a. The vertical timescale as applied in previous studies^18,19^, calculated from Eq. (2) using ${w}_{*}$, is presented too; here the sampling height *z* (here: 240 m) instead of the boundary layer height *h* is used. The magnitudes of the convective timescale *t* and DTW derived timescales are not expected to match, as both timescales consider convective transport differently. The DTW derived time represents mixing within up and subsident downdraft transport in the first 325 m of the ABL. Conversely, *t* represents the timescale of individual convective updrafts within the entire ABL.

It should be noted that the extracted vertical timescale varies in the vertical and is correlated with the turbulent kinetic energy (TKE) timescale shown in Fig. 3b. The timescale of the TKE, obtained from DALES, has a maximum at around half the height of the ABL^25^. The observed layer marked with blue arrows thus comprises the transition to maximum vertical velocity indicating that the mixing time based on DTW has a non-linear dependency on height.

### Sensitivity towards OH chemistry

**Figure 4:**
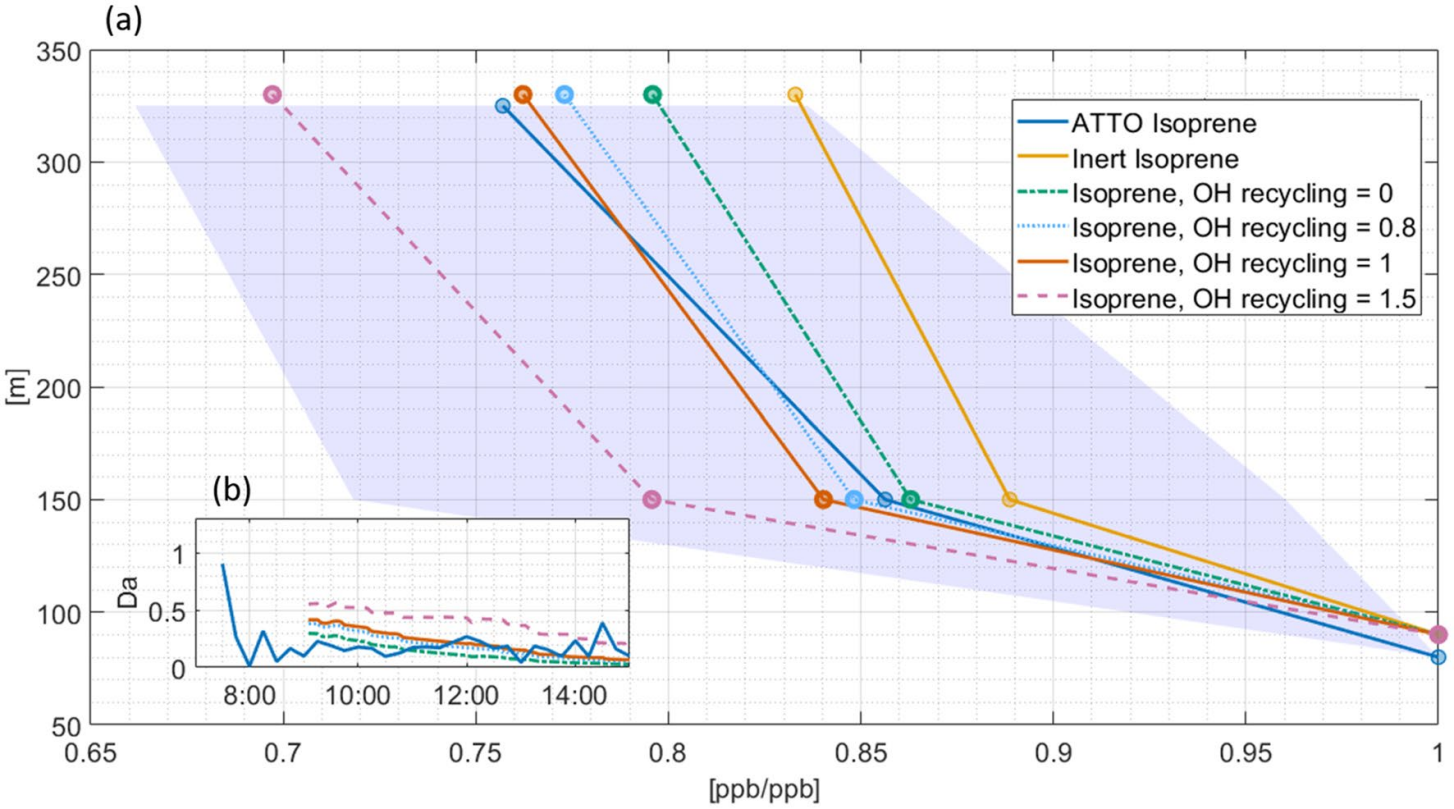
Isoprene profiles above the rainforest canopy under varying OH concentrations simulated with DALES and compared to ATTO observations and the resulting Damköhler Number (Da). (**a**) Relative vertical gradients, median averaged between 12:00–14:00 of isoprene under increasing OH recycling efficiencies calculated with DALES. Inert isoprene represents a non reactive tracer with identical initial profile and emission flux as isoprene. The shadings of the measured isoprene gradient indicate the (0.15, 0.85) quantiles. (**b**) In the box insert the variation of Da for the different recycling efficiencies and over the course of one day is shown. The Dynamical Time Warping based mixing time of DALES is used here. For the Da at ATTO isoprene observations, the mixing time at ATTO (Fig. 3) and final OH concentrations (Fig. 5) are applied.

To better understand the sensitivity of the isoprene concentration gradient in the vertical to OH radical abundance, a suite of systematic experiments that vary OH recycling efficiencies were performed by DALES (Fig. 4). The OH radical recycling efficiency, namely the extent to which OH is regenerated during the oxidative degradation of organic molecules, is a current topic of research especially in low NO_x_–high isoprene regions^41^. Within the BVOC-NO_x_-O_3_-mechanism for gas phase chemistry in DALES (Supplementary Table S1), OH recycling takes place via the reactions of OH + Isoprene → RO_2_ and RO_2_ + HO_2_ → *n*·OH + product, where *n* is a tunable parameter. Figure 4 shows that the best fit to the ATTO isoprene observation was obtained for the DALES model with recycling efficiencies around *n* = 0.8–1, but all cases of recycling are incorporated within the day to day variation of the measurement. The presented simulated vertical isoprene gradients are significantly larger for conditions with increased OH recycling; a one percent increase in OH decreases isoprene by about 0.7—1.2% at 330 m between 12:00 and 14:00 LT. This sensitivity varies throughout the day, and isoprene is less sensitive to a change in OH concentration in the morning. Investigations with DALES reveal that the oxidation of isoprene is likely limited by the availability of isoprene itself, suggesting a higher intensity of segregation occurs in the morning.

To further elucidate the effect of chemistry, a chemically inert analogue of isoprene was also simulated in the DALES model. Diurnal emissions were set to be the same as for isoprene (Supplementary Fig. S2). The resulting residual gradient of the inert isoprene analogue provides evidence for dilution of isoprene emissions by entrainment of isoprene free air from above. The ensemble of up- and downward motions that tend to mix isoprene concentrations and thus counteract the establishment of a vertical gradient also effects the inert analogue of isoprene. Together, this constitutes a relative decrease of the inert isoprene tracer of 16.8%, which is more than half of the total isoprene decrease. A tracer that can be considered here as chemically inert and that is also measured at ATTO is the conserved variable *q*. It is defined as the mass of water vapor in 1 kg of moist air^25^. The annual mean lifetime of water vapor from evaporation to precipitation in the Amazon region is about 3–6 days^42^ so that it can be classified in this context as practically inert. Isoprene and water vapor originate from the canopy or below, and both are diluted with drier and isoprene poorer air from the above layers. Noontime (12:00–14:00 LT) relative decrease of *q* representing the loss due to turbulent mixing was 9.2% (Supplementary Fig. S3a). The (0.15, 0.85) quantiles for the gradient of *q* is 7.9% and 10.2% respectively. Subtracting either the loss of *q* or the simulated inert isoprene from the total decrease in isoprene yields its oxidation driven decrease.

It should be noted that a local effect of cloud shading is that the isoprene emissions can drop abruptly. As a result, there can be momentarily more isoprene aloft than lower down. Combined with inert isoprene or *q*, which are not or less sensitive towards a change in local irradiation, the corrected isoprene gradient can turn positive for short periods, which are not representative for the convective ABL. Those cases result in negative OH concentrations (Supplementary Fig. S4) and are filtered out for both methods. The opposite, an enhanced negative isoprene gradient due to reduced irradiation of the 320 m footprint area is less probable as it covers a much larger area and is therefore less susceptible to complete shading.

Subtracting the gradient of q or of the simulated inert isoprene thus implies an oxidation driven decrease of 18.5% or 15.3% in isoprene at noon, that is used to infer the OH concentration. There is a large difference between the gradients of *q* and inert isoprene which most likely results from different emission and entrainment strengths of H_2_O and isoprene. The emission flux of H_2_O relative to its mixing ratio above, at 90 m, is calculated using DALES and reveals a smaller emission and dilution velocity for *q* compared to inert isoprene (Supplementary Fig. S3b). This can explain the larger gradient of inert isoprene in the investigated layer. Choosing *q* enables correction for each day individually, which is an advantage as the observed gradients of *q* and isoprene show variability between days, whereas the inert isoprene tracer shows one representative day. Also, *q* at 80 m is, like isoprene, influenced by the complex turbulence within the roughness sublayer above the tall canopy. For comprehensiveness and as combining simulated and observed parameters creates additional uncertainty, both ways are applied to infer OH in the following section. However, using simulated inert isoprene is expected to obtain better results as the strength of emission, which is the same for isoprene and inert isoprene, is very relevant in the first 325 m of the ABL.

The diurnal evolution of Da, calculated from Eq. (1) for the five DALES experiments and for ATTO data is presented in Fig. 4b. Values between 0.01 and 0.6 decreasing between 8:00 to 16:00 LT for all OH recycling efficiencies were obtained from DALES. Those values indicate a regime with competing dynamics and chemistry as shown already by the sensitivity of the isoprene gradients towards OH chemistry. At ATTO, the mixing time (Fig. 3) is somewhat faster than the isoprene-OH chemistry lifetime, which ranges from one hour to one day. This is indicated by Da < 1, except for the period in the morning where high OH concentrations lead to fast oxidation chemistry and a Damköhler number of 1.7. The origin of elevated OH concentrations before 8:00 LT is discussed later in the Results and Discussion section. Besides the timescale comparison over the course of one day, a Damköhler number of around one (Da ≈ 1) indicates maximum intensity of isoprene segregation (*I*_s_)^37,38^. The derived values for Da are below one, with a tendency to be closer to one in the morning. To derive *I*_s_, measurements with a higher time resolution than used in this study are necessary. For completeness in the following section a control case with no reduction of the reaction rate constant between isoprene, IsoO and OH is compared to a case with 30% reduction as deduced maximally from a previous study^37,43^. In that study, the deviations from chemical equilibrium were investigated by comparing a turbulence resolving large eddy simulation to a chemistry box model. Recently, Clifton et al.^39^ found a maximum *I*_s_ of 9% for the reaction of isoprene and OH at canopy height that decreases with altitude. They combined a multilayer model for vegetation canopy coupled with in- and above-canopy chemistry and a fine scale large eddy simulation to obtain *I*_s_. Assuming an intensity of segregation of 30% thus represents an extreme case that provides an upper limit for resulting OH concentrations.

### Inferring OH

Two methods were applied to estimate OH. Both are based on the removal of isoprene by OH only (Eq. 3), as its reaction rate coefficient with the second most potent oxidant, O_3_, is about seven orders of magnitude slower and O_3_ levels are below 20 ppb. Kuhn et al.^18^ found a contribution of O_3_ to the depletion of isoprene of 1%. The formula of the first method reads:

3 $$\frac{{\partial \left[ {{\text{Iso}}} \right]}}{\partial t} = - k_{{{\text{Iso}},{\text{OH}} }} \left[ {{\text{OH}}} \right] \left[ {{\text{Iso}}} \right]$$

where $k_{{\text{Iso,OH }}} = 1 \times 10^{ - 10}$ cm molecules^−1^ s^−1^ (IUPAC) is the rate coefficient of the reaction of isoprene and OH. Solving Eq. (3) for OH is a simple but effective way to estimate its concentrations with the observed gradient of isoprene and the estimated reaction time. Effectively air parcels containing isoprene follow the ensemble of up- and downward motions between 80 and 325 m for the duration approached by the derived mixing time. The isoprene concentrations at both heights were detected sequentially, but within a 15 min period. As the transport between the heights is expected to happen on longer timescales, the measurements conducted at 325 m have to be shifted by the respective mixing time to account for turbulent transport and mixing between the heights. This also holds for the observed *q* at 80 and 325 m.

In the previous section the necessity to avoid the erroneous attribution of the dynamical driven reduction of isoprene to OH was demonstrated. Therefore, its partitioning to the isoprene gradient is considered by subtracting the relative decline of *q* or the simulated inert isoprene tracer (9.0% or 15.4% on daily average) from the isoprene gradient. Hereafter this is referred to as the gradient method.

The second method includes the formation as well as further oxidation of IsoO (Eq. 4). Combining and integrating Eqs. (3) and (4) gives a time dependent relation for the ratio of isoprene and IsoO that represents the progress of oxidation^18,19,44^.

4 $$\frac{{\partial \left[ {I{\text{soO}}} \right]}}{\partial t} = y_{{{\text{IsoO}},{\text{OH}} }} \frac{{\partial \left[ {{\text{Iso}}} \right]}}{\partial t} - k_{{{\text{IsoO}},{\text{OH}}}} \left[ {{\text{OH}}} \right] \left[ {{\text{IsoO}}} \right]$$

5 $$\frac{{\left[ {{\text{IsoO}}} \right]}}{{\left[ {{\text{Iso}}} \right]}} = y_{{{\text{IsoO}},{\text{OH}} }} \frac{{k_{{{\text{Iso}},{\text{OH}} }} }}{{k_{{{\text{IsoO}},{\text{OH}}}} - k_{{{\text{Iso}},{\text{OH}} }} }} \left( {1 - e^{{\left( {k_{{{\text{Iso}},{\text{OH}}}} - k_{{{\text{IsoO}},{\text{OH}} }} } \right) \left[ {{\text{OH}}} \right] t}} } \right)$$

where $y_{{{\text{IsoO}},{\text{OH}} }}$ is the total yield of MVK, MACR and ISOPOOH from the oxidation of isoprene^45^ and $k_{{{\text{IsoO}},{\text{OH}} }}$ is the rate coefficient of IsoO + OH. The chemical partitioning of IsoO strongly depends on the availability of NO_x_ as MVK and MACR are formed in a second step via NO while ISOPOOH emerges from the reaction of RO_2_ and HO_2_^46^. At ATTO the portion of ISOPOOH depends not only on the abundance of NO but also possibly on wall exchange effects of the sticky ISOPOOH in the inlet line (< 330 m)^47^. As this potentially generates considerable uncertainty the effect of a varying ISOPOOH proportion on the resulting OH was considered by two cases of 100% and no line loss for ISOPOOH. Assuming no line loss the proportion of ISOPOOH constitutes 50% of the IsoO signal in accordance with Rivera Rios et al.,^45^ for NO concentrations of 50 ppt, whereby MVK + MACR both contribute 25%. Consequently, the weighted yield is $y_{{\text{IsoO,OH }}} = 0.7$ and the weighted reaction rate is $k_{{{\text{IsoO}},{\text{OH}}}} = {5}.{6} \times {1}0^{{ - {11}}}$ cm molecules^−1^ s^−1^. Without considering ISOPOOH the weighted yield is $y_{{{\text{IsoO}},{\text{OH}} }} = 0.{55}$ and $k_{{{\text{IsoO}},{\text{OH}}}} = {2}.{35} \times {1}0^{{ - {11}}}$ cm molecules^−1^ s^−1^. Equation 5 was then discretized and integrated with a time resolution of 1 s. By comparing the evolved time with the derived mixing time, the height can be allocated and OH is adjusted to fit the observed ratio of isoprene and IsoO at 325 m. This method is referred to as the ratio method. Here again, the isoprene concentration gradient is corrected for its reduction due to dynamical processes by subtracting the gradient of *q* or inert isoprene. A limitation of the ratio method is that dynamic processes affecting the IsoO gradient and its simultaneous formation via isoprene oxidation cannot be separated, thus cannot be corrected for. IsoO is formed most effectively when isoprene concentrations are highest, above the canopy, but mixing of IsoO in the up- and downdrafts and further formation modify the resulting gradient. Also, possible entrainment of IsoO poor air during the ABL growth may influence its gradient. In that respect it would be ideal to have observations available above the ATTO tower to account for this. Thus, it is possible that the IsoO gradient due to chemistry only may be stronger than the observed gradient, especially when turbulent mixing is strong. In conclusion, OH calculated with the ratio method can be expected to be an underestimate.

**Figure 5:**
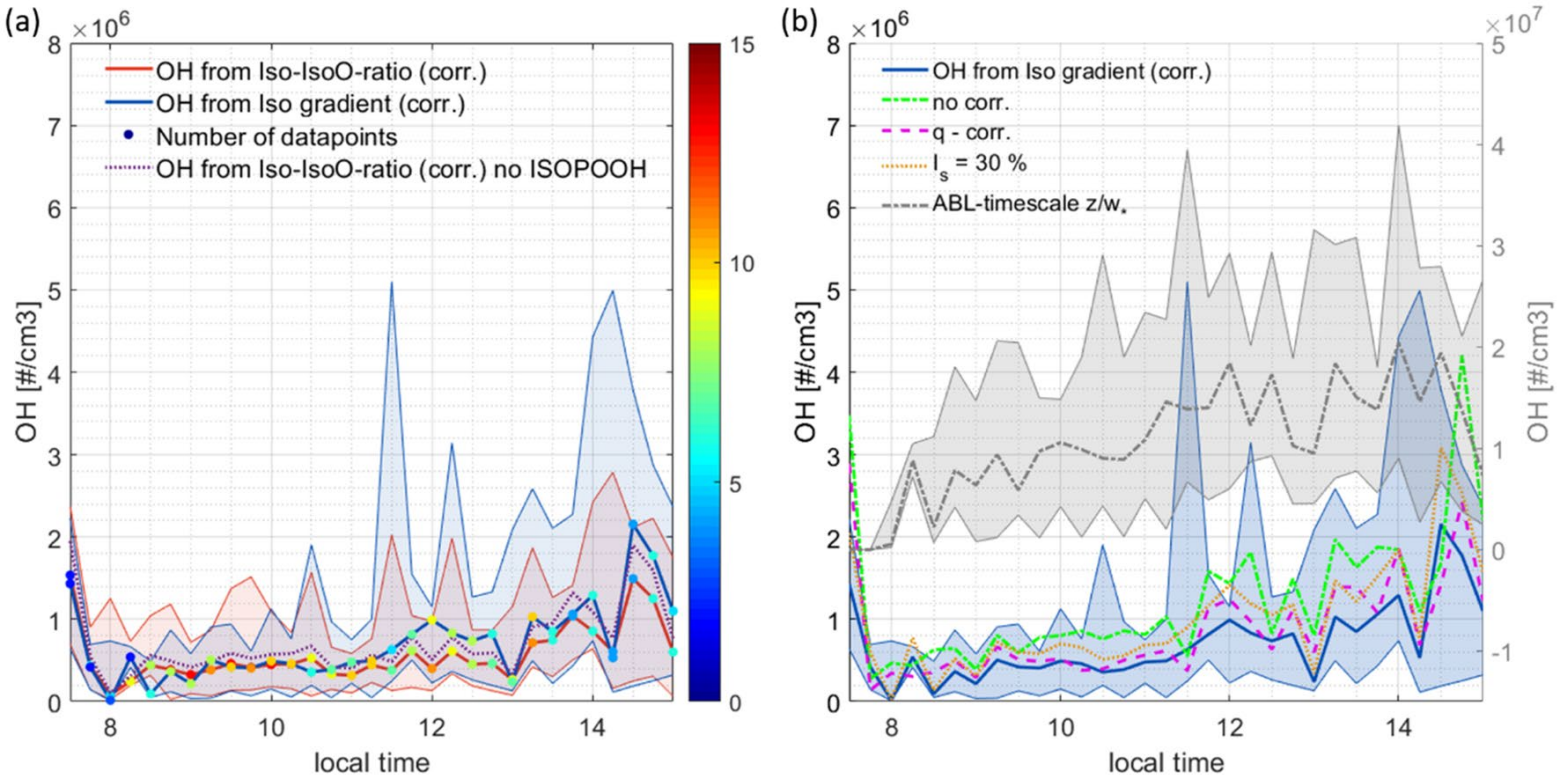
OH concentrations inferred over the course of one day from ATTO observations. The shadings indicate the (0.15, 0.85) quantiles. (**a**) The daily evolution of estimated OH using the gradient and ratio based method corrected for the impact of dynamics on the isoprene gradient using the inert isoprene simulated by DALES. The color code represents the number of median averaged days. The sensitivity of OH towards the composition of IsoO without ISOPOOH is shown in purple. (**b**) The plot includes sensitivity of OH from the gradient method towards the Damköhler effect, the reaction time used and the correction for the effect of dynamics with inert isoprene (blue) and *q* (pink). OH concentrations inferred with the ABL-timescale (grey) have units on the right axis.

Uncertainty in both methods is implied by further factors, such as the composition of IsoO, the segregation of isoprene, turbulent transport and the reaction time in the vertical. These are addressed in Fig. 5. The measurement uncertainty is estimated and provided in the methods section.

The OH concentrations thus derived (Fig. 5a) yield median average values ((0.15, 0.85) quantiles) between 8:00 and 15:00 LT of 5.3 (2.0, 12.7) × 10^5^ molecules cm^−3^ and 4.5 (1.6, 11.8) × 10^5^ molecules cm^−3^ for the isoprene gradient based method and the ratio-based method respectively. The enhanced OH concentrations before 8:00 LT are not considered in the average as they represent very few observations, but are discussed separately in the next section. OH derived with the first method ranges from 0.2 (0.1, 7.4) × 10^5^ to 2.2 (0.2, 3.8) × 10^6^ molecules cm^−3^ at around 14:00 LT. Here, the simulated inert isoprene was used to correct for dynamics affecting the isoprene gradient. After 11:30 the Iso-IsoO-ratio method indicates lower OH concentrations than the isoprene gradient method likely due to the limitation of the IsoO gradient with respect to mixing and entrainment. We therefore conclude that the isoprene gradient method gives more realistic OH concentrations as dynamic processes are considered for the isoprene gradient. Both methods have a large day-to-day variation due to the limited numbers of observations. It should be noted that fewer data are available in the early morning and late afternoon due to the application of the filters to ensure convective conditions.

The importance of applying the mixing time, derived by DTW, rather than the convective velocity ${w}_{*}$ of individual updrafts becomes clear when comparing the resulting OH estimates, see Fig. 5b. OH concentrations from the latter method exceed the OH estimate of this study 19-fold on average throughout the day with values around 1.0 (0.4, 2.0) × 10^7^ molecules cm^−3^. The correction for dynamic processes with inert isoprene simulated by DALES, as described above, is greatest after 12:00 when turbulent transport is very efficient. Its OH concentrations yield 54% of the uncorrected OH concentrations averaged over one day. Compared to the correction using *q* it yields 79% of the OH throughout the day. However, OH concentrations using *q* or no correction are mainly found within the (0.15, 0.85) quantiles of the OH values using the inert isoprene tracer. Next, the isoprene and IsoO segregation effect on OH, when assumed to be at the upper limit of *I*_s_ = 30%, leads to 42% higher OH concentrations than derived with no effect of segregation but still lies within its day-to-day variation. Finally, the IsoO composition that encompasses between 0 and 50% ISOPOOH has a non-negligible impact on the ratio method, with OH concentrations for the case with 50% ISOPOOH reaching 79% of OH inferred for 0% ISOPOOH (IsoO = 50% MACR + 50% MVK) (Fig. 5a). When taking into account all uncertainties of the gradient method, using an average correction for dynamic processes of 16.8% derived from the inert isoprene tracer, the upper limit of the method is achieved by assuming *I*_s_ = 30% which constitutes a rather extreme case. The upper limit of the inferred OH concentrations ranges from 0.2 (0.1, 1.1) × 10^5^ to 3.1 (0.3, 5.4) × 10^6^ molecules cm^−3^ (Fig. 5b).

Previous OH measurements conducted in the atmosphere-canopy interface region of forested sites or at the forest edge with little anthropogenic influence are presented in Table 1. They are in general agreement with the OH concentrations estimated here when considering the high isoprene and low NO conditions observed at ATTO. The OH reactivity measured at ATTO corresponds most closely with the SOAS campaign in the southeastern US, although the SOAS site had lower isoprene and higher NO abundance^48^. SOAS reported a reactivity of up to 35 s^-1^ about 5 m above the canopy while Pfannerstill et al. 2021 measured up to 40 s^−1^ at 50 m above canopy at the ATTO site during the dry season of 2019 parallel to the observations used in this study^32^. OH concentrations up to 2 × 10^6^ molecules cm^−3^ measured during SOAS also agree well within the variability of the OH inferred in this study. Both the lower isoprene and higher NO is expected to enhance SOAS OH relative to ATTO, however, photolysis rates at the tropical ATTO site are higher. Another OH measurement was made in the Amazon rainforest during GoAmazon, with OH concentrations in the same range although the simulated OH reactivity was substantially smaller and the measurements were performed at ground level next to the forest^49^. Conversely, during GoAmazon concentrations of O_3_, which is involved in OH recycling under low NO_x_ conditions ^1^, exceeded the concentrations detected at ATTO, being below 0.15 ppb on average at 38 m height just above the canopy. Similar levels of OH have been observed at midlatitudes where production rates and BVOC fluxes are both lower ^50^. In the boreal forest where insolation is lower and BVOC fluxes are high the OH concentrations are correspondingly lower, but still 3–6 × 10^5^ molecules cm^−3^^51^. All OH concentrations detected in forested, remote sites in the interface region of the canopy and the atmosphere would be overestimated using ${w}_{*}$ to approximate the reaction time, whereas the mixing timescale yields comparable results. A separate study using a variability-lifetime based method to indirectly estimate OH from airborne measurements of nonmethane hydrocarbons in the first km of the ABL in Surinam reported average OH concentrations of 2 × 10^5^ molecules cm^−3^^52^.

**Table 1:** Overview of OH observations conducted in largely remote, forested sites.

Project	Year	Site	Height above ground [m]	OH measured (#/cm^3^)	NO (ppt)	O_3_ (ppb)	Isoprene (ppb)	OH (s^−1^)	
PROPHET	1998/2008	Michigan, USA	31	1.9–3.9 × 10^6^/0–3 × 10^6^ ^a^ (FAGE)	< 150^a^	30–47^a^/22–35^a^	1–2.5/ 0.2–2.5^a^	–	^11, 53^
GABRIEL	2005	Surinam	< 1000 (ABL)	5.6 × 10^6^ ± 1.9^b^ (FAGE)	20 ± 20^b^	18.5 ± 4.6	2.00 ± 0.76^b^	–	^6^
OP3	2008	Borneo	6 (forest edge, canopy height 10 m)	0–2.5 × 10^6 a^ (FAGE)	20–200^a^	5–15^a^	0–3^a^	5-30^a^	^54^
CABINEX	2009	Michigan, USA	31	0–1.5 × 10^6a^ (FAGE)	< 150 ^a^	22–35^a^	0.1–2^a^	2–14^a^	^53^
SOAS	2013	Alabama, USA	15 (canopy height 9-12 m)	0.2–2 × 10^6 a^ (LIF)/0–2 × 10^6 a^ (CIMS)	< 300^a^	15–40^a^	1–7^a^	15–33^a^ (LIF) /18–35^a^ (CIMS)	^48^
GoAmazon	2014/2015	Amazonia, Brazil (T3)	2 (forest edge)	0.2–1.5 × 10^6 a^ (CIMS)	0–150^a,c^	21^d^	0.5–3^a^	8.5^e^	^40, 49^

^a^Values taken from averaged daily cycles. ^b^Daily mean values. ^c^Box model simulations with MCM v3.3.1. ^d^Mean values 10:00–15:00 LT. ^e^Simulated mean values 10:00–15:00.

All previous measurements tabulated above show a diel cycle in the averaged OH concentrations with a maximum around solar noon, driven by its photolytic source. This is also observed in this study with maximum OH occurring around 14:00 LT. For the studies presented in Table 1 the maximum OH concentration ranges from 11:00–15:00 LT. Interestingly none of the previous observations reported a second OH maximum (1.4 × 10^6^ molecules cm^−3^) in the morning as was the case from both methods applied in this study.

### OH simulated using a hierarchy of models: turbulence-resolved, regional and global

**Figure 6:**
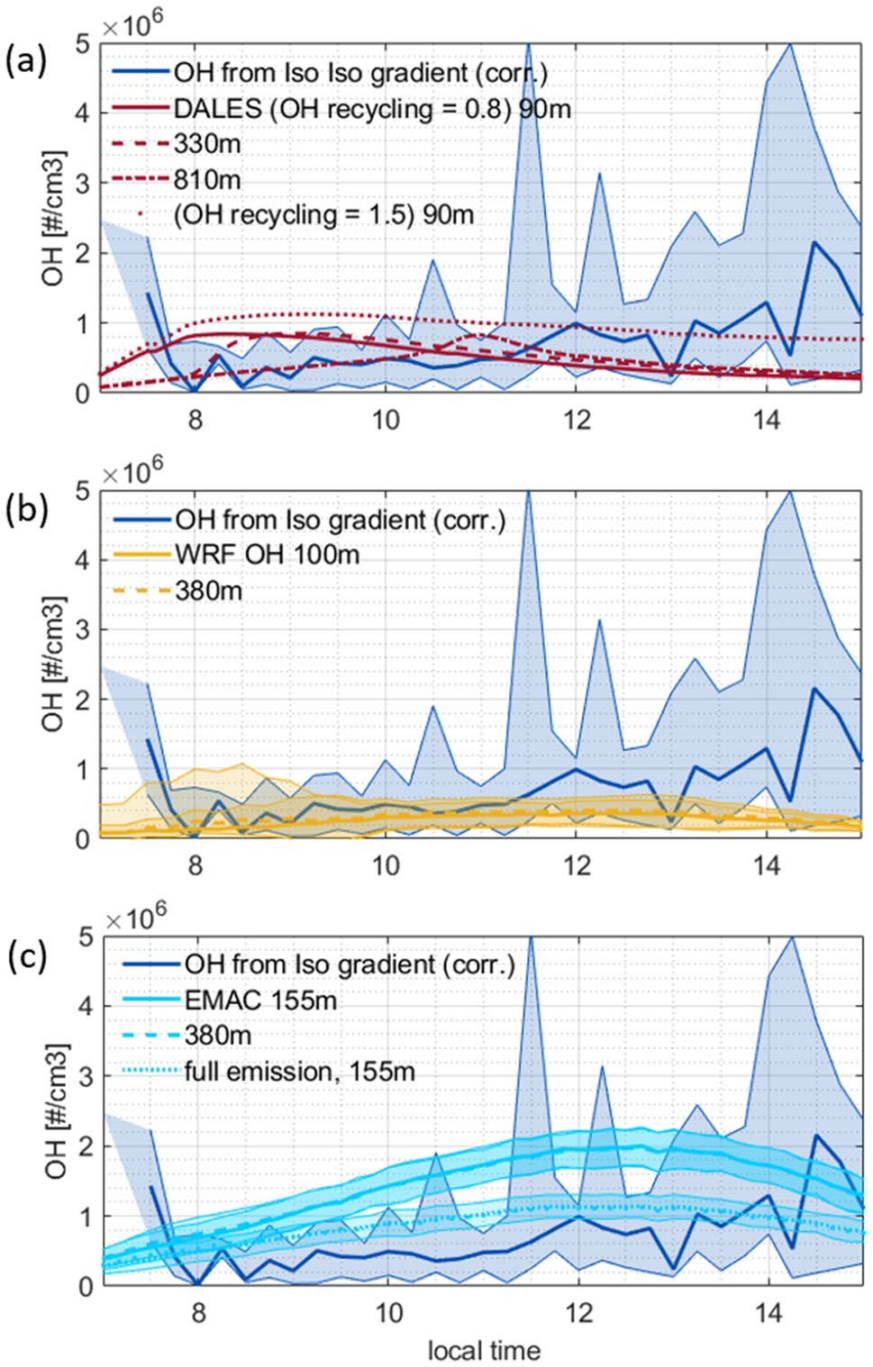
Diurnal variability of OH time series at different heights compared to OH estimate in this study (80–325 m) with the gradient method. The shadings indicate the (0.15, 0.85) quantiles for inferred OH and 1 sigma for modeled concentrations. (**a**) DALES results are obtained by including the sensitivity towards OH recycling efficiencies of 0.8 and 1.5 (**b**) WRF-Chem (**c**) EMAC run with halved biogenic emissions at different heights and the full emission for comparison.

Three different models were used to simulate OH radical concentrations as a function of time-of-day and height above forest, and these were compared to the OH inferred in this study. Results from the turbulence-resolving transport model DALES (50 × 50 × 20 m), a nested regional atmospheric chemistry model WRF-Chem (3 × 3 km, 7 layers in the first 500 m), and a global atmospheric chemistry model EMAC (208 × 208 km, 3 layers in the first 500 m) are shown for the dry season in Fig. 6. In the case of DALES one typical day is simulated whereas EMAC and WRF-Chem present median average time series of the dry season in 2010 and 2018 respectively, with precipitation events being as well excluded.

Model simulations were matched with the estimated OH from the isoprene gradient method, which was deemed to be most reliable (see section “Inferring OH”). Large differences in the predicted OH concentration and its daily variability are found between models. The highest OH concentration up to 2 × 10^6^ ± 0.3 × 10^6^ molecules cm^−3^ is predicted by the global model EMAC which shows a classical diurnal evolution following the intensity of solar irradiation and photochemistry. The diurnal cycle of WRF-Chem has a similar shape exhibiting a second early morning maximum on some days at 380 m, but relatively low concentrations up to 3.6 × 10^5^ ± 1.8 × 10^5^ molecules cm^−3^ around noon. Up until about 11:30 LT WRF-Chem produces OH concentrations similar to those of the gradient method. Simulated OH by DALES shows highest values in the morning at 90 m and 330 m depending on the OH recycling efficiency. Choosing *n* = 1.5 from the DALES runs that were performed to test the sensitivity of the isoprene gradients towards OH, fits best the averaged empirically estimated concentrations. Though it overestimates concentrations derived before 11:30 LT and underestimates the values thereafter as it shows decreasing OH concentrations throughout the day. Looking at higher altitudes in DALES reveals a shift of the daily maximum towards noon. The two major differences between the models is the spatial resolution around the location of ATTO and the complexity of chemistry. DALES is equipped with a modest chemistry scheme (Supplementary Table S1) but high-resolution simulation (20 m) of vertical turbulence; WRF-Chem uses the larger MOZART-4 gas phase chemical mechanism scheme^55^ and a medium spatial resolution, while EMAC involves the very detailed MECCA^56^ chemistry scheme but has a coarse vertical and horizontal resolution.

**Figure 7:**
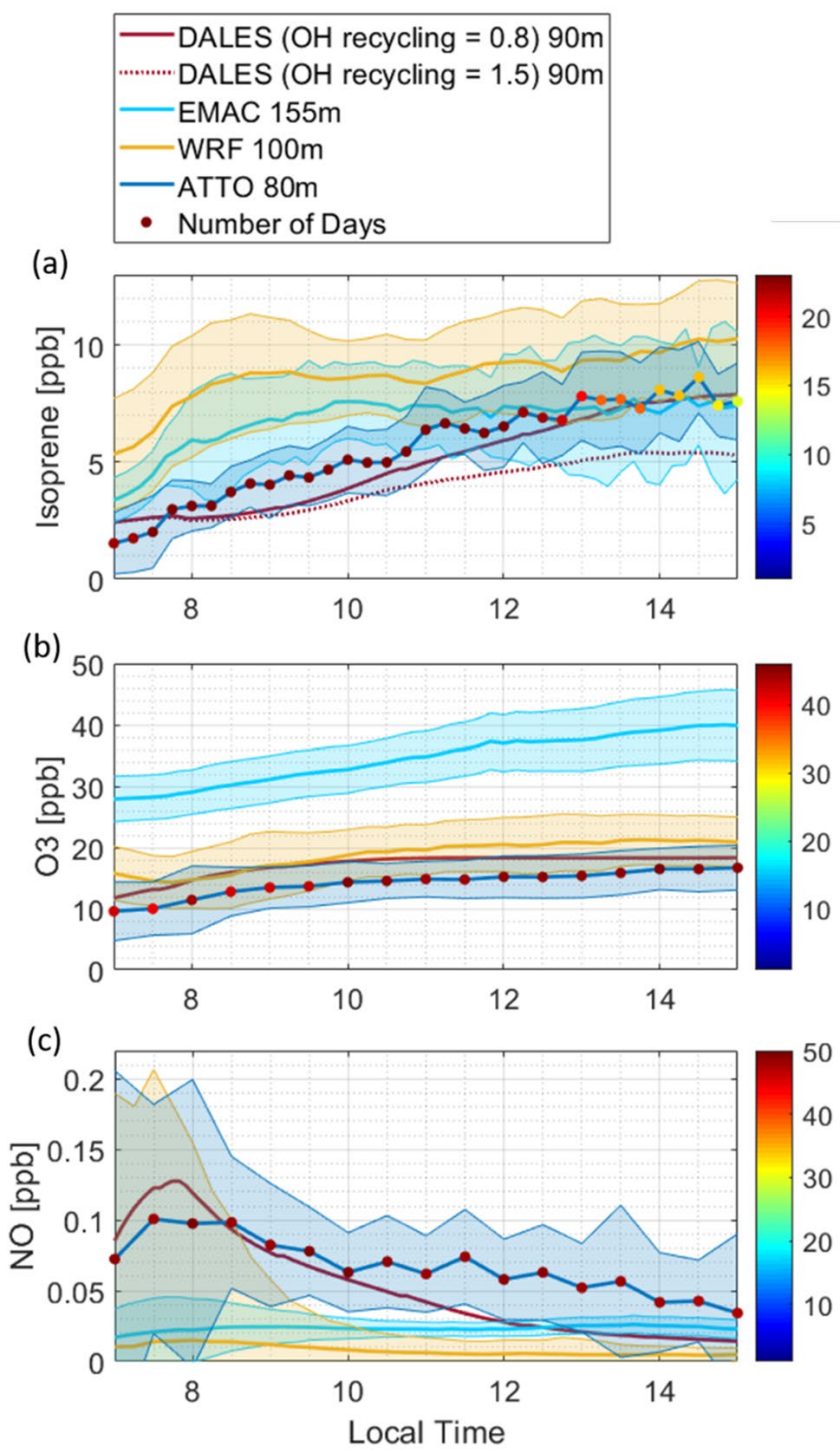
Diurnal variability of simulated Isoprene, O_3_ and NO concentration time series compared to ATTO observations under no-rain conditions. The shadings indicate the (0.15, 0.85) quantiles for observations and 1 sigma for modeled data. (**a**) Isoprene mixing ratio measured at ATTO compared to model results. The number of days with isoprene data presented as colored marks decreases after noon as precipitation events are excluded. For DALES simulations with two different recycling efficiencies are included. (**b**) O_3_ observed and simulated (**c**) NO observed and simulated.

Due to a highly parameterized vertical exchange in EMAC, local issues occur, e.g. the temperature at the canopy interface is too high so that a large amount of isoprene and other BVOC with temperature dependent emission parameterizations is emitted from surface vegetation^57^ (Supplementary Fig. S5). To overcome the mismatch, the EMAC isoprene emissions are arbitrarily reduced by about half, to avoid passing the issue on to all mechanisms involving isoprene^58^. Figure 6 also includes the case of full isoprene emissions which depress the OH concentration substantially. Nevertheless, the reduced isoprene emissions are still higher compared to DALES and WRF-Chem. In Fig. 7 the isoprene mixing ratios of the three models are compared, as well as dominant driving factors for OH like O_3_ and NO. Taking them into account, high OH concentrations in EMAC are a result of high O_3_ and recycling within the MECCA chemistry scheme. Elevated O_3_ concentrations in EMAC are suspected to arise from a strong entrainment from above layers due to the highly parameterized vertical transport.

The O_3_ concentrations of WRF-Chem and DALES are closer to mixing ratios observed at ATTO, but in WRF-Chem average NO is additionally very low. The standard deviation of NO from WRF-Chem is enhanced in the morning between 6:00 and 9:00 LT though, when also DALES and ATTO measurements show peaking NO from nocturnal accumulation of soil emission and subsequent ejection into the ABL during its transition to a convective layer. The reaction of HO_2_ with NO to form OH^13^ leads to temporally enhanced OH concentrations between 6:00 and 9:00 LT which can be seen in the empirically inferred OH and for DALES as well as for WRF-Chem on some days. In the case of DALES this is the only maximum during the day, as the production of OH via NO is the largest source of OH over the day (Supplementary Fig. S6). The chemistry scheme of DALES does for example not include terpenes other than isoprene which are considered in the detailed chemistry schemes of WRF-Chem and EMAC, so differences in sources and sinks for OH are generated (Supplementary Fig. S7). Further investigation of the DALES simulated OH showed that the diurnal evolution and the morning peak in particular depends strongly on the initial boundary conditions of NO_x_. These initial profiles depend on the chemistry and the emissions occurring in the nocturnal boundary layer and in the residual layer before sunrise. The empirical method has the advantage that entrainment of NO_x_ from the residual layer and formation of OH is inherently considered. Therefore, it can act to guide the large eddy simulation along with additional observations.

DALES and WRF-Chem produce elevated OH concentrations due to NO between 8:00 and 9:00, but at ATTO the OH peaks before 8:00 LT reaching 1.4 × 10^6^ molecules cm^−3^. On average observations of NO in the 2018 dry season peak at 7:00–9:00. However, data coverage is limited to only four days of overlapping inferred OH and NO data. These days all show enhanced NO before 8:00 LT (Supplementary Fig. S8). The elevated OH concentrations at ATTO shortly after sunrise are found on two days, which stand out because the temperature profiles indicate unstable conditions before 8:00 LT, earlier than usual, connected with long mixing times and high isoprene gradients observed (Supplementary Fig. S9). Thus, the elevated OH concentrations between 7:00 and 8:00 LT could be strongly influenced by the morning transition and the boundary layer growth if air at 325 m is still affected by the isoprene poor nocturnal stable boundary layer. This is prevented by regarding the same airmass at 80 and 325 m, ensured by the shift of 325 m isoprene concentrations according to the mixing time. High OH concentrations derived with the ratio method might also be caused by an enhanced increase of IsoO with height. Entrainment of IsoO rich air from above the ATTO tower, accumulated in the nocturnal boundary layer can not be ruled out without further measurements at higher levels. Another possible explanation is that the accumulated NO ascends together with the emitted isoprene earlier than on the other considered 21 days. To evaluate the ratio- and gradient-method during the boundary layer transition in closer detail more parallel measurements of NO and VOC will need to be acquired.

## Conclusion

A novel and measurement-constrained method for the empirical estimation of the diurnal variability of OH radical abundances has been developed and applied to data obtained from the 325 m ATTO tower in the remote Amazon rainforest. In this region, high fluxes of BVOC coincide with high primary production rates of OH^1^, highly variable vertical mixing and low NO concentrations. Under these conditions, uncertainties regarding OH recycling mechanisms have been reported to be significant^49,59^. Our findings show that the resulting daytime median averaged OH concentrations are characterized by peaks in the morning (short) and after solar noon (main), varying between 8:00–15:00 LT from 0.2 (0.1, 7.4) × 10^5^ to 2.2 (0.2, 3.8) × 10^6^ molecules cm^−3^. OH concentrations within that part of the day are consistent with observations previously conducted at rainforest sites. The peak in the morning needs to be verified by additional simultaneous observation of NO and isoprene to infer OH concentrations during the nocturnal-diurnal transition over of a tall rainforest canopy. Furthermore, the reaction of isoprene and OH under different mixing conditions with regard to the intensity of segregation needs to be known to reduce the OH variability uncertainty in the morning.

To infer accurate OH variations over the day, we show the need to consider emission, turbulent mixing and dilution of the isoprene by entrainment to adequately quantify the partitioning of these dynamic processes and the oxidative chemistry taking place simultaneously. By introducing the DTW-derived mixing time (105–15 min) based on observations of the warming of the lower ABL, we find that the timescales of mixing are faster than isoprene chemistry but within a similar range throughout the day. The choice of the reaction time estimation has a large impact on the resulting OH concentration. Here, the assumption of using a timescale based on the convective velocity ${w}_{*}$ inferred from individual convective updrafts needs to be revisited. The DTW derived mixing time represents the time shift due to the observed warming between two levels, driven by vertical motions and lateral mixing. The plumes of air containing isoprene and OH also undergo lateral mixing and the ensemble of turbulent motions. Accounting for these turbulent motions as a whole is crucial when analyzing measurements in the roughness sublayer or lower boundary layer. This is substantiated by the agreement of observed and estimated OH concentrations under similar environmental conditions when applying the mixing time, whereas applying ${w}_{*}$ yields concentrations one order of magnitude higher.

Comparing the diurnal variability of OH inferred in this study against a hierarchy of models, all characterized by different assumptions in resolving turbulent transport (explicit or parameterized), vertical and horizontal resolution and in chemical mechanisms, we find that all applied models provided very different OH concentrations and temporal patterns in the first 380 m. Greater agreement of the diurnal variability between the models was achieved above 500 m. We therefore conclude that the lower interface region characterized by a tall canopy is still difficult to model accurately, being complex in both chemistry and dynamics. The dynamical time warping method presented here has been shown to be suitable for calculating mixing times and hence OH concentrations and it can be applied when direct OH observations are lacking. In future it will be of great interest to compare our estimates to in-situ OH measurements if they can be realized at the site. Since this region governs emission export to, and oxidant levels in, the free troposphere, further measurements and numerical models that explicitly account for the canopy-atmosphere interactions are recommended to examine OH characteristics in other seasons and ecosystems.

## Method

### Observation

The observations presented here were made at the Amazon Tall Tower Observatory (ATTO), a collaborative Brazilian-German project, located 135 km north-east of Manaus (02.14°S,58.99°W, 120 m above sea level). A detailed description of the observatory including a map is published in Andreae et al., 2015 ^17^. A detailed map of the ATTO site can be found in the supplementary (Supplementary Fig. S15). The site provides close to pristine conditions with low levels of NO_x_ in the absence of nearby athropogenic sorces^60^. A Proton Transfer Reaction Time of Flight Mass Spectrometer (PTR-TOF–MS; Ionicon Analytik, Innsbruck, Austria)^61^ was installed within an air-conditioned laboratory container at the foot of the ATTO tower. Sample inlets (3/8″OD insulated Teflon) are installed from the container to 80 m, 150 m and 325 m, so that semi-continuous measurements can be made at three heights by sequentially sampling at each height for 5 min. Each height is therefore sampled for 5 min every 15 min. The time resolution was 20 s averaged to 1 min. The PTR-TOF–MS was operated with hydronium ions (H3O +) at a pressure of 3.5 mbar and an E/N of 120 Td. VOC mixing ratios were obtained by calibrating to a VOC gas-standard (Apel-Riemer Environmental Inc., Colorado, USA). Isoprene (m/z 69.069) can be detected as low as 40 ppt, with an uncertainty of 11.6% and a precision of 3.2%. The oxidation products of isoprene, measured as their sum at m/z 71.049, MVK, MACR and ISOPOOH can be detected with a total uncertainty of 12.3% and 2.3% precision down to 21 ppt. The total uncertainty includes the precision and the calibration error. Possible line loss of those compounds is in experience neglected. In this study, 23 days of data from the dry season (September and October) of 2018/2019 is used under exclusion of periods with precipitation for the use of DTW (in 2018: 10/21/2018–10/24/2018 and in 2019: 09/01–09/06, 09/08–09/16, 09/18, 09/19, 09/21 and 09/26). Median mixing ratios for isoprene were found to vary from 1.48 ± 0.35 ppb after sunrise up to 8.12 ± 1.92 ppb at 14:30 at 80 m height averaged over the considered period. IsoO follows the daily evolution of isoprene with median values of 1.05 ± 0.21 ppb to 4.00 ± 0.80 ppb at 80 m. The mixing ratios measured represent the integrated emission of the forest biosphere, which is assumed to be uniform over the whole catchment area of ATTO^17^. Meteorological and flux data up to 80 m is measured at 80 m on the Instant tower, a walk-up-tower in 1 km distance from ATTO^17^ (LI7500A, LI-COR Biotechnology, Lincoln,USA) and at a weather station at 325 m installed directly on the ATTO tower (Lufft, WS600-LMB, G. Lufft Mess- und Regeltechnik GmbH, Fellbach, Germany). Both meteorological datasets are available with a 1 min time resolution. The potential temperature has a measurement uncertainty of less than 1% at 80 m and 325 m. For the period studied here, there was no meteorological data available for the altitudes in between 80 and 320 m. To estimate the uncertainty of OH due to the precision of the measurement of isoprene, IsoO and *θ,* a Monte Carlo simulation is performed for the gradient and the ratio method. Samples with 10,000 normally distributed random values for each variable (isoprene, IsoO, mixing time) constrained by the median and the precision of the measurement are used to calculate OH and its distribution. The uncertainty of the mixing time related to the DTW method is small, but uncertainty may arise from local anomalies that alter *θ* only on one of the two sample heights. For the purpose of demonstration, it is assumed to be 0, 5 and 10%. We report uncertainty factors relative to the median, derived from the 15% and 85% quantiles since the distribution of OH concentrations is not normal. For the gradient method uncertainties of ${\text{OH}}_{0.59}^{0.88}$ assuming no uncertainty of the mixing time, ${\text{OH}}_{0.59}^{0.90}$ for 5% uncertainty of the mixing time and ${\text{OH}}_{0.59}^{0.91}$ for 10% uncertainty of the mixing time are estimated. The calculation of OH with the ratio method is computationally more demanding, thus a sample number of 2,000 is applied for the Monte Carlo simulation. It yields ${\text{OH}}_{0.41}^{0.50}$ for 10% uncertainty on the mixing timescale. The OH variability with measurement uncertainty is shown in the Supplementary Fig. S16.

### Atmospheric models

The model applied is the numerical experiment, described by Vilà‐Guerau de Arellano^40^ based on the Dutch Atmospheric Large-Eddy Simulation (DALES)^21^ with an integrated O_3_‐NO_x_‐VOC‐HO_x_ chemical system. The numerical experiment is designed according to observations made during the GoAmazon campaign in September 2014 (dry season), that took place in Amazonia^62^. The initial profiles of isoprene and its oxidation products are constrained by the observation regarded in this study, therefore they are set to 2 ppb and 0.2 ppb for all altitude levels respectively. Wind is calculated explicitly using Navier–Stokes simulations. Surface winds that induce mechanical turbulence and wind profiles are compared to sounding observations with good agreement^40^. DALES has a vertical resolution of 20 m and a horizontal resolution of 50 m so that the dimension of the ATTO tower is well represented in the model. The forest canopy is treated as a single layer. At the high of the measurements 80 m and 325 m, the coupling of vegetation and atmosphere processes, including the interaction with clouds, vegetation responses to radiation and meteorological conditions and turbulent transport during the course of the day is explicitly (not parameterized) reproduced by DALES. The large eddy simulation code and the numerical experiment settings can be accessed here: http://doi.org/10.5281/zenodo.3759193.

The Weather Research and Forecasting coupled with chemistry (WRF-Chem)^22^ is a regional atmospheric chemistry model which has been extensively used in the past for several regions of the world for a variety of studies requiring high spatial and vertical resolution^63,64^. By setting up nested domains such that the outer-most (parent) domain is driven by a chemical and meteorological boundary conditions of a global model or global reanalysis dataset, one can achieve spatial resolutions of up to a few km.

For the current study, WRF-Chem was set up in a nested configuration such that spatial resolution of the parent domain (d01) is 15 × 15 km^2^ and that of the inner domain (d02) is 3 × 3 km^2^. Vertically the setup has 42 terrain following layers from surface until 50 hPa with 3 and 10 layers in the bottom 80 m and 1 km, respectively. The simulation was performed for a period between 01/09/2018 and 20/10/2018. The meteorological boundary conditions were prescribed by the ERA5 reanalysis dataset^65^ updated every 3 h. Similarly, the chemical conditions were prescribed from the archived model output of CAM-Chem every 6 h available from https://www.acom.ucar.edu/cam-chem/cam-chem.shtml. We also employed spectral nudging of geopotential height (ph) and U and V components of wind from ERA5 above the boundary layer height to make sure that the large scale features of the simulations don’t drift away from the reanalysis dataset while allowing it to resolve the fine scale meteorological features^66^.

Biogenic and soil NO_x_ emissions in WRF-Chem are calculated online using the MEGAN version 2.0, while the anthropogenic and fire emissions are prescribed from the EDGAR v5.0 inventory^67^ and GFAS^68^ respectively. We used the HERMESv3 tool^69^ for mapping of the emissions inventories on the WRF-Chem horizontal grid.

The ECHAM/MESSy Atmospheric Chemistry (EMAC) is the Earth System Model with core atmospheric general circulation model ECHAM5^70^ coupled with the Modular Earth Submodel System (MESSy) implementing relevant (sub)grid processes (trace gas/aerosol chemistry, emission, dry/wet removal of species, etc.) in one consistent modelling framework^23,24^. In the current study we use the output of the MESSy v2.54 evaluation study^58^ performed with a model resolution T63L31 (equivalent to 1.88° or about 208 km at the ATTO location in horizontal, model top at 10 hPa or approx. 31 km). In comparison to DALES and WRF-Chem, EMAC has coarser spatial resolution (three lowermost layers span approximately 500 m with interfaces at about 70 and 250 m) and more parameterized representation of the BL dynamics and tracer transport^71^, yet being equipped with the most comprehensive kinetic chemistry mechanism MOM^1,72^. The latter intrinsically reproduces high OH reactivities over the Amazon^1^ however isoprene emissions reduced by 50% of their nominal strength (calculated by the MEGAN v2.1 model^73^) yield best agreement with the observed OH and isoprene surface concentrations globally and at ATTO^58^. Similar to WRF-Chem, EMAC atmospheric dynamics are nudged towards the ERA5 [ref.above] reanalysis fields, the anthropogenic and fire emissions of NO_X_ and BVOC are calculated based on the EDGAR v4.3.2 and GFAS v1.0 inventories, respectively. Model output (grid column encircling ATTO location, no interpolation used) for September 2010 from the simulated 2008 − 2017 period is analysed here.

### Dynamical time warping

DTW is a method to allocate common points of two differently stretched curves in order to warp one of the curves into the other ^20^. It was developed within the field of speech recognition in order to identify words that are the same but pronounced differently. It is, however, applicable to many forms of time-series. In a first step the daily time-series are normalized to values between zero and one. Then a two-dimensional cross-distance-matrix $D=d\left(x,y\right)$ containing the Euclidean distance between every pair of elements is created (Supplementary Fig. S10). $x$ and $y$ are the time indices of each time series respectively. The goal of DTW is to find the best allocation, that is the path through the cross-distance-matrix that yields a minimal distance between elements of the time-series. The so-called warping path defines possible allocations and underlies certain constraints ^74^:

- 1. The boundary conditions can force the warping path to start or end in the opposite diagonal corners of the cross-distance-matrix.
- 2. To ensure continuity the warping path must proceed continuous through the cross-distance-matrix
- 3. The warping path must be monotonic to avoid pointless detours and loops
- 4. A user defined windowing function forbids the warping path to cross the window (Supplementary Fig. S10) that prevents negative mixing times $\left(x\left(i\right)-y\left(i\right)\ge 0\right)$
- 5. User defined step patterns can constrain the steps taken by the warping path, e.g. it must not move straight upwards, leading to a warping path with unique indices $y.$ In this study the ‘symmetric2’ step pattern is used, that allows all steps that fulfil the points 1–4.

The warping path reallocates the time indices $x$ and $y$ according to the minimalized warping path. The result is an allocation of the data points of two timeseries, which is presented in Fig. 2. The warping yields the relative temporal variabilities between both curves, which shows in this application how one curve is followed by the other. The last step is to calculate this time shift, here the mixing time, from the obtained allocation. The time indices correspond to the temporal resolution of the time-series, so Eq. (6) results in the time shift for every element of the time-series.

6 $$t_{mixing} = \left( {x\left( i \right) - y\left( i \right)} \right)*res_{t} ,i = 1 \ldots n$$

$n$ is the length of the array of indices. With the applied settings is possible that multiple indices $x\left(i\right)$ are allocated to one index of $y\left(i\right)$, if this is the case the average of the distances $x\left(i\right)-y\left(i\right)$ give the mixing time. The property used to derive the mixing time needs to fulfill two requirements: It must have primary (emission) source located at the level of the rainforest, and its chemical lifetime must strongly exceed the mixing time. We chose the potential temperature (*θ*) to be used to obtain the mixing time because conductive heating takes place at the ground and at canopy level leading to lateral mixing of up and downdraft turbulent motions and thus to heating of the ABL during convective conditions at daytime. *θ* is also indifferent towards chemical processing. The resulting time shift is the consequence of boundary layer warming through the mixing of heat fluxes.

### Supplementary Information

Supplementary Information 1.

Supplementary Information 2.

## Data Availability

Profile data of VOC [ppb] that support the findings of this study will be available in the ATTO Data Portal via https://doi.org/10.17871/atto.300.3.1213. Meteorological data and products, pressure, temperature, relative humidity and z/L conducted at the Instant tower (80 m) that support the findings of this study have been deposited in the ATTO Data Portal and are accessible upon request at http://attodata.org/. Meteorological data, pressure, temperature and relative humidity conducted at the ATTO tower (320 m) in 2019 that support the findings of this study are available via https://doi.org/10.17871/atto.95.12.742. For the year 2018 this data is deposited in the ATTO Data Portal and accessible upon request at http://attodata.org/. Profile data of NO and O_3_ [ppb] that support the findings of this study have been deposited in the ATTO Data Portal and are accessible upon request at http://attodata.org/. Outputs from DALES, WRF-chem and EMAC that support the findings of this study are available from J. Vilà-Guerau de Arellano, V. Kumar and S. Gromov respectively upon reasonable request.

## Code availability

Dynamical Time Warping (version 1.22–3) is a freely available package for R (version 3.6.1). Commented R code is attached (Supplementary Information 2).
