# Supplementary material for: Inferring the diurnal variability of OH radical concentrations over the Amazon from BVOC measurements
Source: Sci Rep. 2023 Sep 9;13:14900. doi: 10.1038/s41598-023-41748-4 (PMC10492859; doi:10.1038/s41598-023-41748-4)
Supplement: Supplementary file 1 — Supplementary Information 1. [file 41598_2023_41748_MOESM1_ESM.docx]

Inferring the diurnal variability of OH radical concentrations over the Amazon from BVOC measurements

A. Ringsdorf^1,*^, A. Edtbauer^1^, J. Vilà-Guerau de Arellano^1,2^, E. Y. Pfannerstill^1a^, S. Gromov^1^, V. Kumar^3^, A. Pozzer^1^, S. Wolff^1^, A. Tsokankunku^1^, M. Soergel^1b^, M. O. Sá^4^, A. Araújo^5^, F. Ditas^6c^, C. Poehlker^6^, J. Lelieveld^1,7^, J. Williams^1,7,*^

^1^Department of Atmospheric Chemistry, Max Planck Institute for Chemistry, Mainz, Germany

^2^Meteorology and Air Quality Section, Wageningen University, Netherlands

^3^Satellite remote sensing group, Max Planck Institute for Chemistry, Mainz, Germany

^4^Instituto Nacional de Pesquisas da Amazônia (INPA), CEP 69067-375, Manaus, Brazil

^5^Empresa Brasileira de Pesquisa Agropecuária (Embrapa) Amazonia Oriental, CEP 66095-100, Belém, Brazil

^6^Department of Multiphase Chemistry, Max Planck Institute for Chemistry, Mainz, Germany

^7^Climate and Atmosphere Research Center, The Cyprus Institute, 1645 Nicosia, Cyprus

^a^now at: Department of Environmental Science, Policy, and Management, University of California, Berkeley, 94720, CA, USA

^b^now at: Friedrich-Alexander-Universität Erlangen-Nürnberg, Sachgebiet Arbeitssicherheit, Erlangen, Germany

^c^now at: Hessian Agency for Nature Conservation, Environment and Geology, Wiesbaden, Germany

*Jonathan.Williams@mpic.de, A.Ringsdorf@mpic.de

Supplementary

**Table S1:** Daytime chemical reaction scheme applied in DALES. The reaction rate coefficients for first order reactions [s-1] and second order reactions [cm^3^ modelcules^-1^ s^-1^] are presented in the right column with *T* representing the air temperature [K]. The rate coefficient of R17 is shown below the table. Species in brackets are chemically inactive. The *n* is a variable describing the recycling efficiency by giving the number of OH molecules emerging from the reaction R19. R1 and R6 are reactions depending on the photolysis of the respective species.

| Number | Chemical equation | Reaction rate constant |
| --- | --- | --- |
| R1 | O_3_ + (*hv)* $\to$ O^1D^ + O_2_ | $3.83\times{10}^{-5}\cdot e^{-0.575}$ |
| R2 | O^1D^ + H_2_O $\to$ 2OH | $1.63\times{10}^{-10}\cdot e^{\frac{60}{T}}$ |
| R3 | O^1D^ + N_2_ $\to$ O_3_ | $2.15\times{10}^{-11}\cdot e^{\frac{110}{T}}$ |
| R4 | O^1D^ + O_2_ $\to$ O_3_ | $3.30\times{10}^{-11}\cdot e^{\frac{55}{T}}$ |
| R5 | NO_2_ + (O_2_) $\to$ NO + O_3_ | $1.67\times{10}^{-2}\cdot e^{-0.575}$ |
| R6 | CH_2_O + (*hv*) $\to$ HO_2_ | $1.47\times{10}^{-4}\cdot e^{-0.575}$ |
| R7 | OH + CO $\to$ HO_2_ + (CO_2_) | $2.40\times{10}^{-13}$ |
| R8 | OH + CH_4_ $\to$ CH_3_O_2_ | $2.45\times{10}^{-12}\cdot e^{\frac{-1755}{T}}$ |
| R9 | OH + ISO $\to$ RO_2_ | $1.00\times{10}^{-10}$ |
| R10 | OH + MVK $\to$ HO_2_ + CH_2_O | $2.40\times{10}^{-11}$ |
| R11 | OH + HO_2_ $\to$ H_2_O + (O_2_) | $4.80\times{10}^{-11}\cdot e^{\frac{250}{T}}$ |
| R12 | OH + H_2_O_2_ $\to$ H_2_O + HO_2_ | $2.90\times{10}^{-12}\cdot e^{\frac{-160}{T}}$ |
| R13 | HO_2_ + NO $\to$ OH + NO_2_ | $3.50\times{10}^{-12}\cdot e^{\frac{250}{T}}$ |
| R14 | CH_3_O_2_ + NO $\to$ HO_2_ + NO_2_ + CH_2_O | $2.80\times{10}^{-12}\cdot e^{\frac{300}{T}}$ |
| R15 | RO_2_ + NO $\to$ HO_2_ + NO_2_ + MVK + CH_2_O | $1.00\times{10}^{-11}$ |
| R16 | OH + CH_2_O $\to$ HO_2_ | $5.50\times{10}^{-12}\cdot e^{\frac{125}{T}}$ |
| R17 | 2HO_2_ $\to$ H_2_O_2_ + (O_2_) | k* |
| R18 | CH_3_O_2_ + HO_2_ $\to$ *Product* | $4.10\times{10}^{-13}\cdot e^{\frac{750}{T}}$ |
| R19 | RO_2_ + HO_2_ $\to$ *n*OH + *Product* | $1.50\times{10}^{-11}$ |
| R20 | OH + NO_2_ $\to$ HNO_3_ | $3.50\times{10}^{-12}\cdot e^{\frac{340}{T}}$ |
| R21 | NO + O_3_ $\to$ NO_2_ + (O_2_) | $3.00\times{10}^{-12}\cdot e^{\frac{-1500}{T}}$ |

* k = (k_1_ + k_2_)k_3_, k_1_ = $2.2\times{10}^{-13}\cdot e^{\frac{600}{T}}$, k_2_ = $1.90\times{10}^{-33}\cdot e^{\frac{980}{T}}\cdot c_{air}$, k_3_ =$1+1.40\times{10}^{-21}\cdot e^{\frac{2200}{T}}\cdot c_{H_{2}O}$


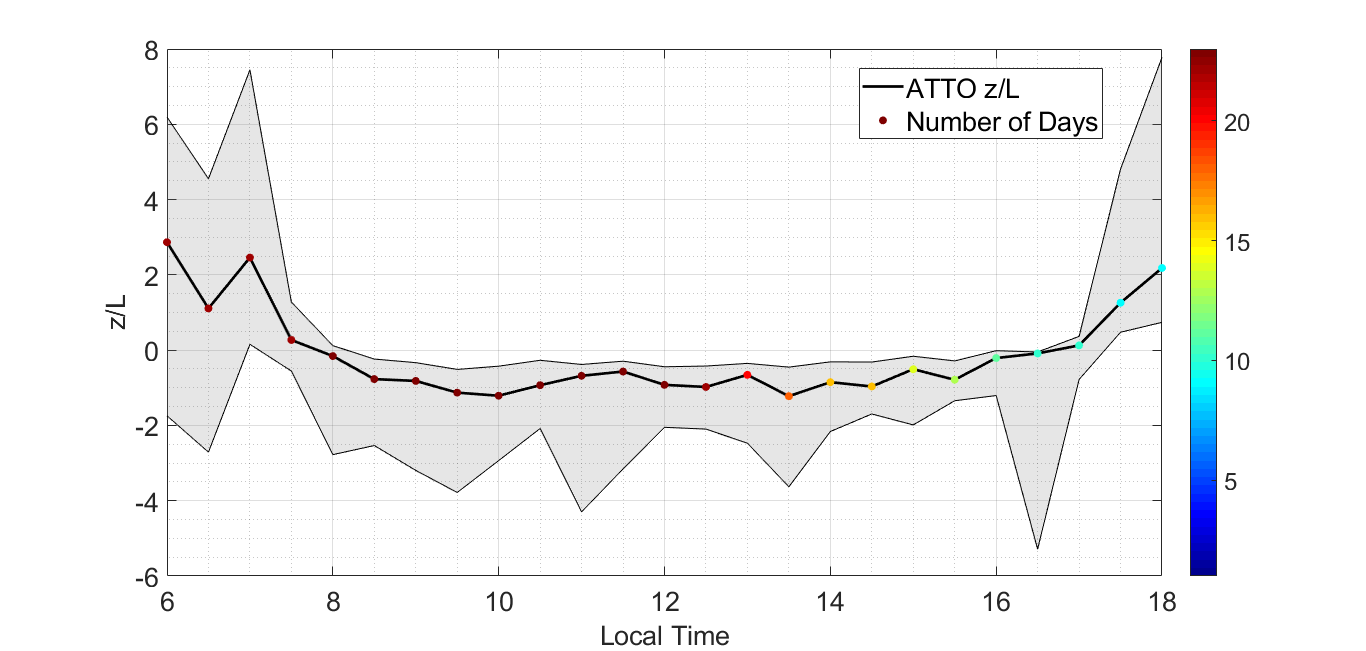


**Figure S1:** Stability parameter z/L observed at ATTO and median averaged over the 23 days used in this study. Precipitation events are excluded. Shadings indicate the (0.15, 0.85) quantiles.


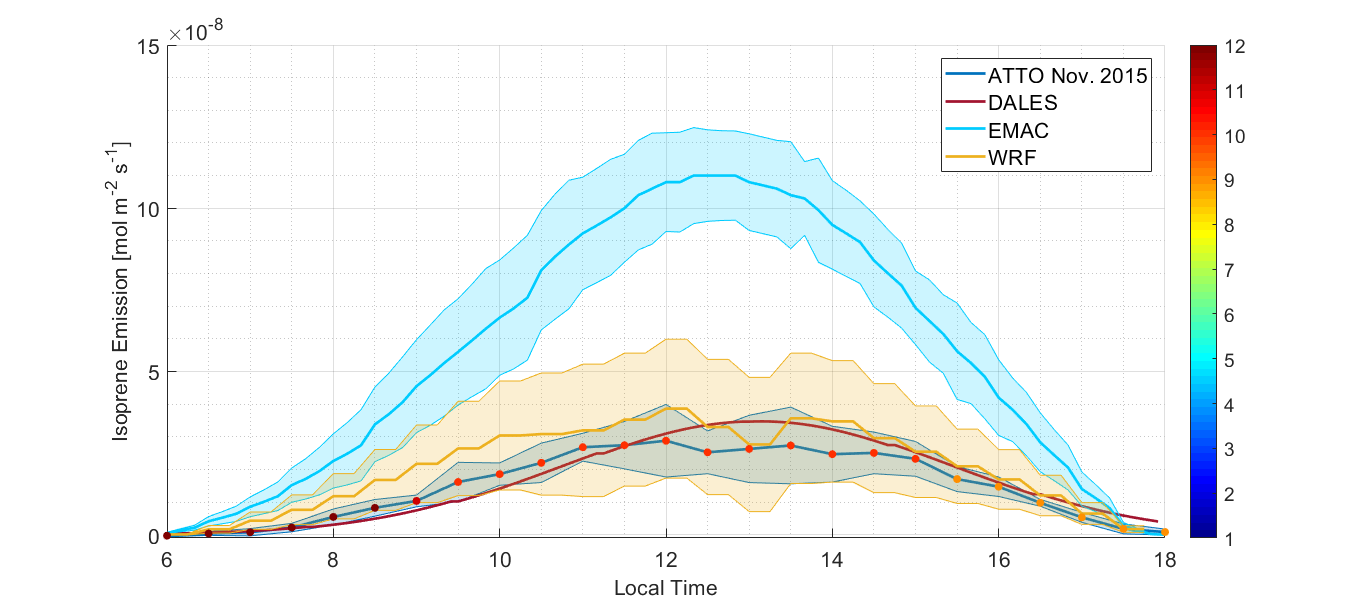


**Figure S2:** Isoprene emission in the numerical models DALES, EMAC and WRF-Chem. For EMAC only the case with reduced isoprene emission is shown. ATTO observations published by Pfannerstill et al. 2018 (<https://doi.org/10.3389/ffgc.2022.952123>) are conducted in the dry season in November 2015. The Number of measurement days is indicated by colored dots. Shadings indicate 1σ.


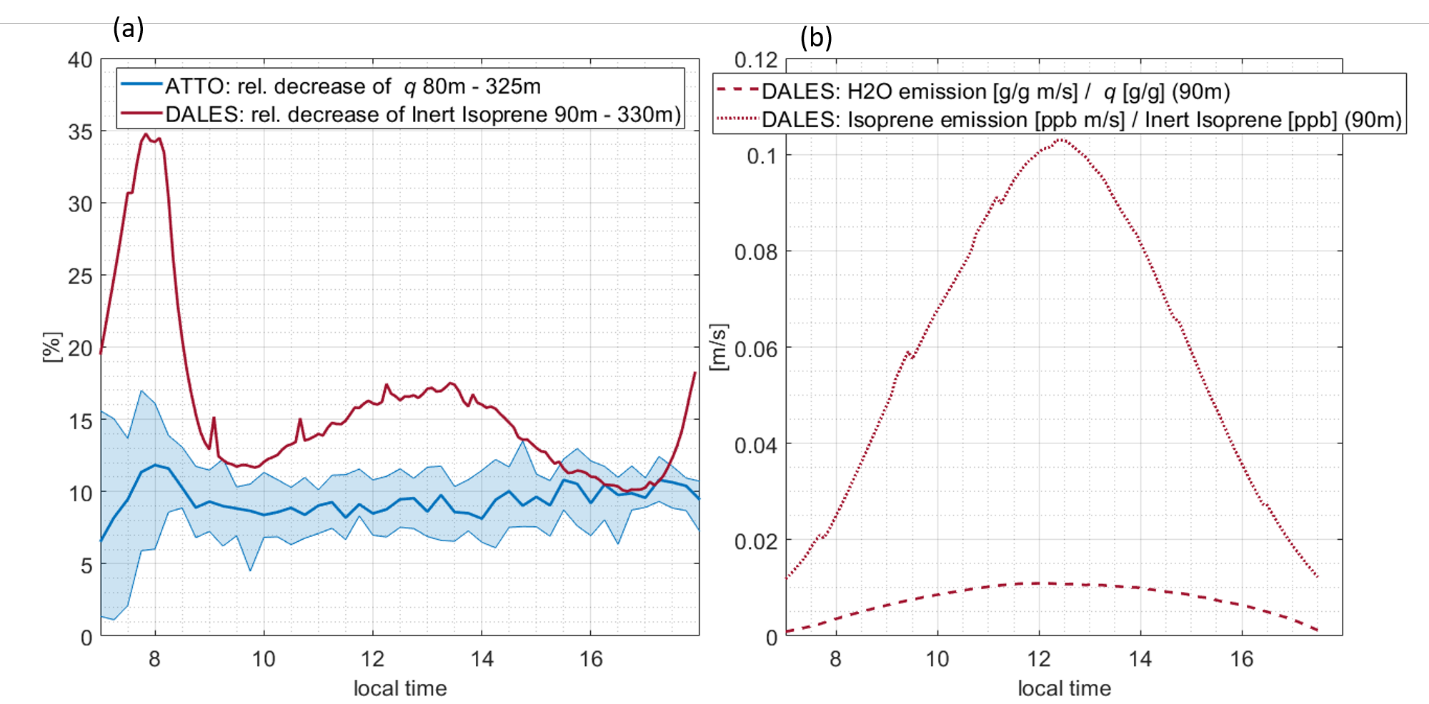


**Figure S3: Comparison of the inert isoprene tracer to q, both used to correct for turbulent mixing** a) The relative decrease of q measured at ATTO compared to inert isoprene simulated by DALES. The shading indicate the (0.15, 0.85) quantiles b) The emission and dilution velocity for q and inert isoprene both calculated from DALES simulations.


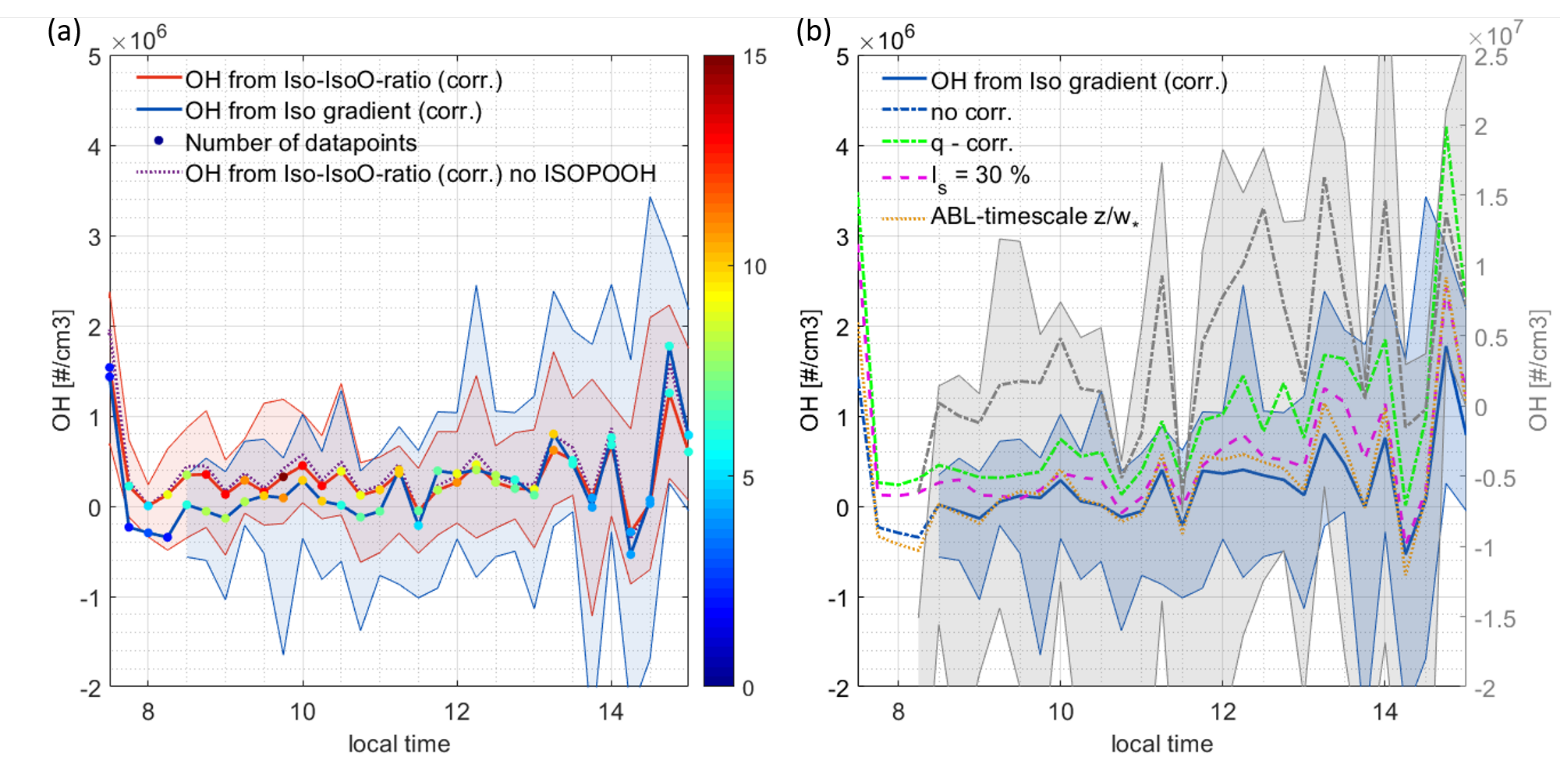


**Figure S4: OH concentrations inferred over the course of one day from ATTO observations including negative isoprene gradients after correcting for dynamic processes. The shadings** **indicate the (0.15, 0.85) quantiles.** **a)** The daily evolution of estimated OH using the gradient and ratio based method corrected for the impact of dynamics on the isoprene gradient using the inert isoprene simulated by DALES. The color code represents the number of median averaged days. The sensitivity of OH towards the composition of IsoO without ISOPOOH is shown in purple. **b)** The plot includes sensitivity of OH from the gradient method towards the Damköhler effect, the reaction time used and the correction for the effect of dynamics with inert isoprene (blue) and q (pink). OH concentrations inferred with the ABL-timescale (grey) has units on the right axis.


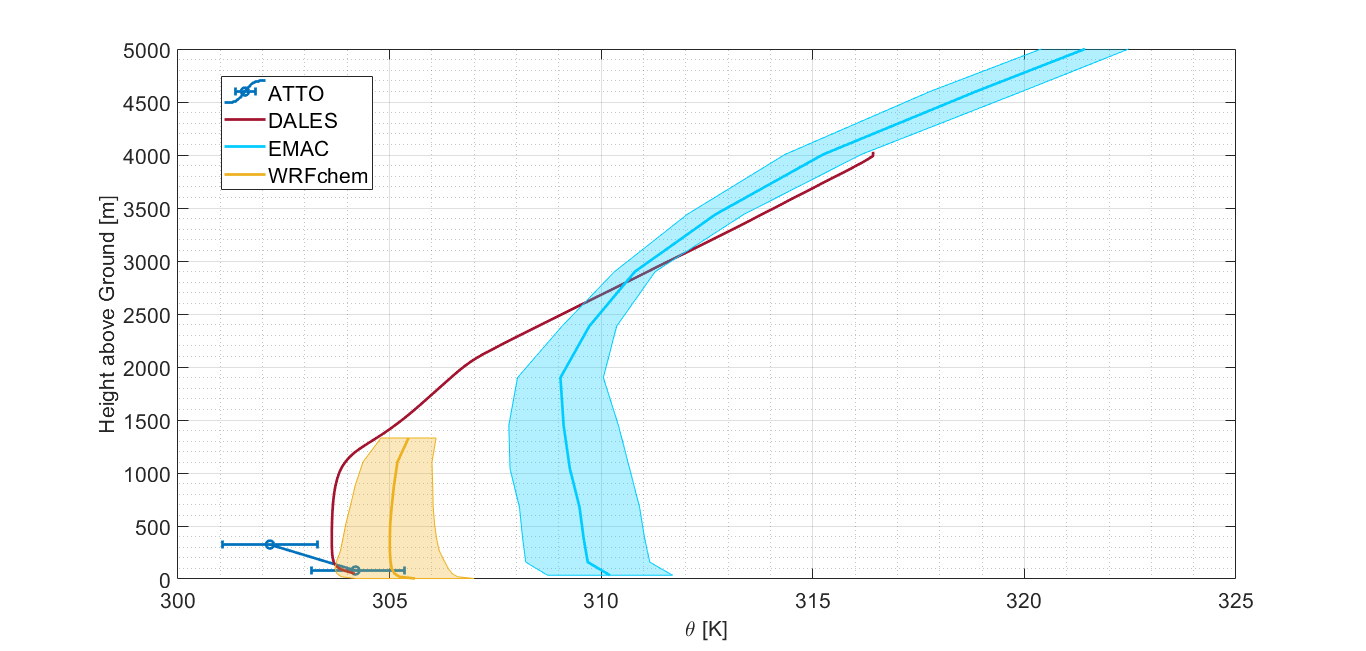


**Figure S5:** The vertical profile of the potential temperature θ for the numerical models DALES, EMAC and WRF-Chem and 1σ variation compared to median averaged observations at ATTO at two heights. Errorbars of the observation indicate the (0.15, 0.85) quantiles.


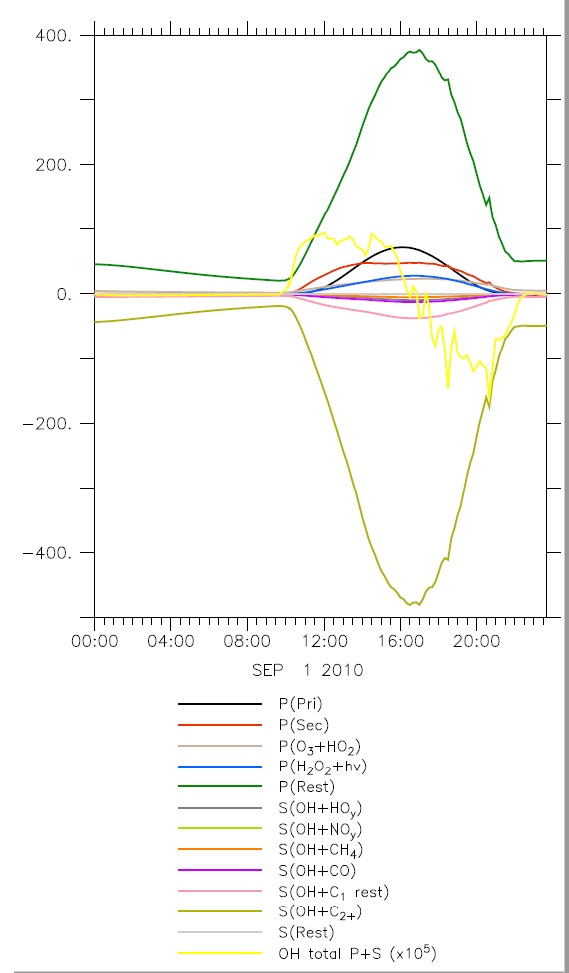


**Figure S6:** Sources and sinks of OH from the global model EMAC at 80 m height extracted for September, 1^st^.


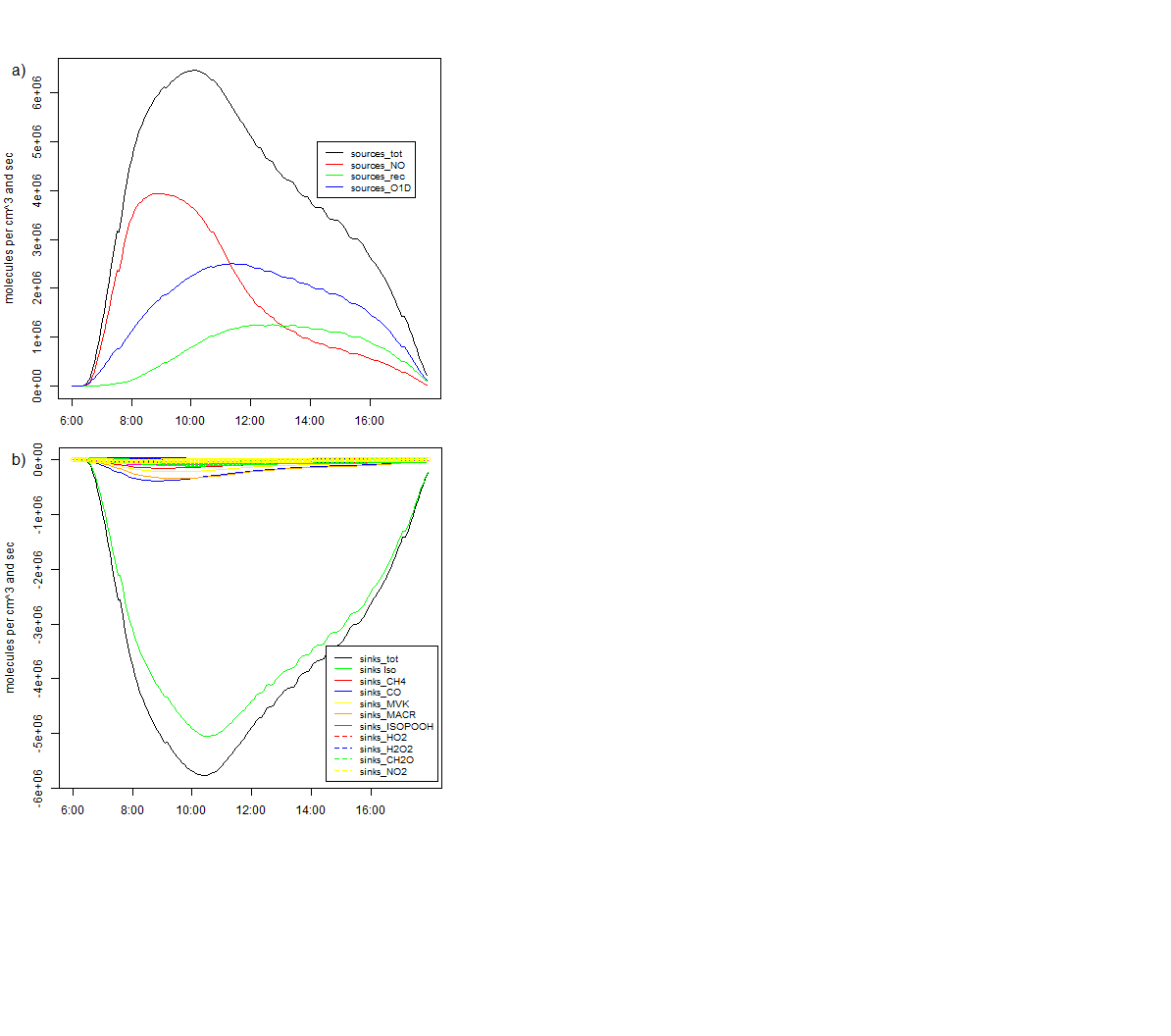


**Figure S7:** **Sources and sinks of OH from DALES over one typical day (local time) of the Amazon dry season at 90 m height.** **a)** sources of OH **b)** sinks of OH


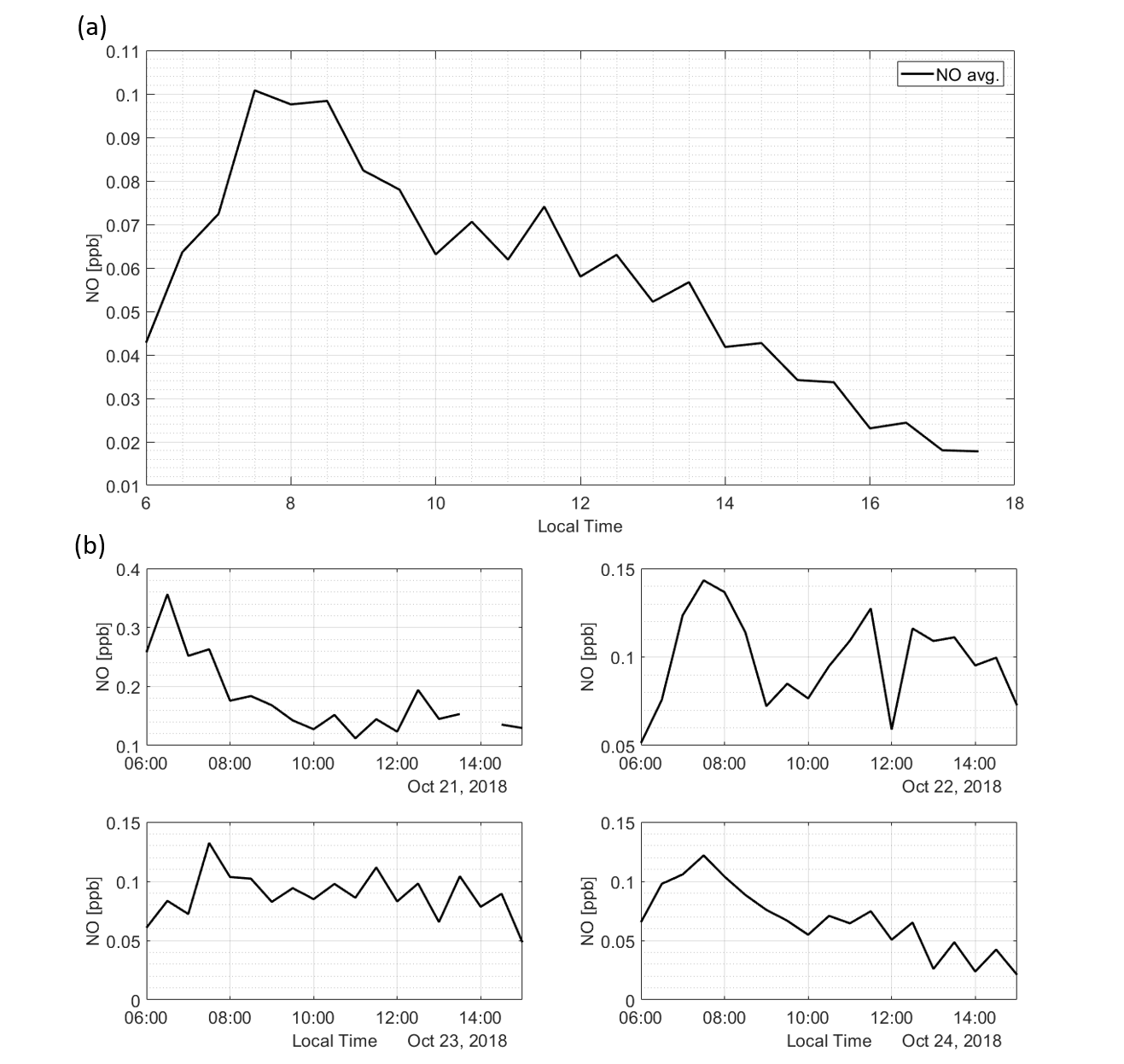


**Figure S8:** **Daily cycles of NO detected at ATTO** **a)** Median averaged mixing ratio of NO measured in the dry season of 2018 (September, October) **b)** The NO mixing ratio measured at ATTO on four days that are considered for the OH estimation as well. All other days have no overlapping data.


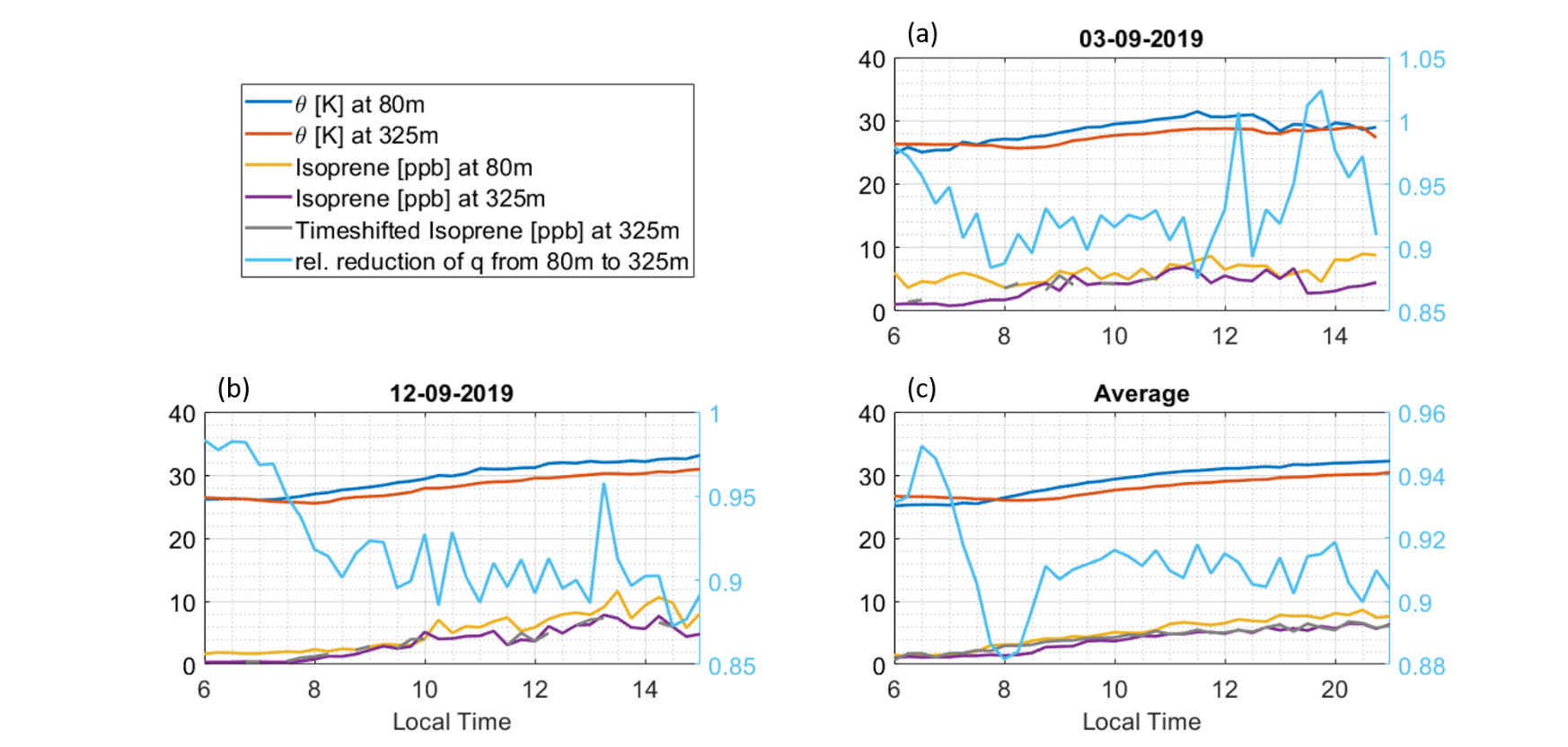


**Figure S9:** Time series of potential temperature θ, isoprene and the relative gradient of q [$\frac{{kg}_{w}/{kg}_{d}}{{kg}_{w}/{kg}_{d}}]$with labels on the right axis on the two days that result in an enhanced OH before 8:00 LT compared to the average. The OH concentration is estimated in the parts of the day when θ at 80 m exceeds θ measured at 325 m, which indicates unstable stratification of the boundary layer. **a, b)** Two days that show a local maximum in OH concentration before 8:00 LT **c)** The average over all 23 dry season days


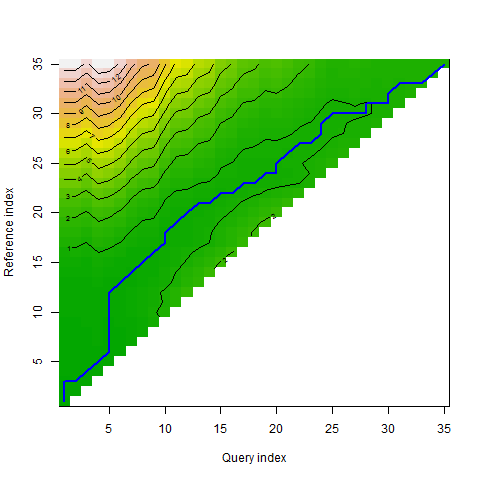


**Figure S10:** Example of a warping path of the Dynamical Time Warping analysis with the positive window cut off. Reference and Query Indexes are the time steps of potential temperature θ at 80 m and 325 m.


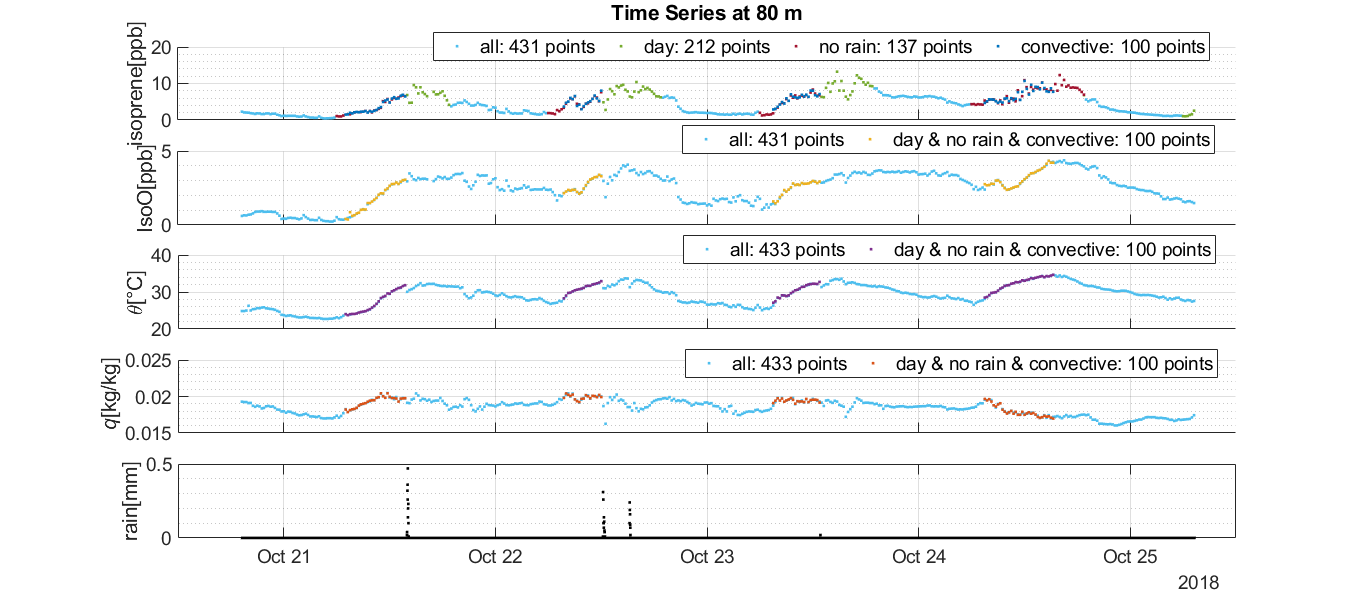


**Figure S11:** Timeseries for the measured data in the dry season 2018 of all variables at 80 m used to calculate OH concentrations. The data is averaged to 15 min resolution as applied in this study. “All” refers to the whole period with simultaneously measured isoprene, IsoO, θ, q and precipitation. “day”, “no rain” and “convective” represent the filter periods for daytime, periods with no rain (and NaN) for longer than 6 hours and convective boundary layer conditions with a negative vertical gradient of θ.


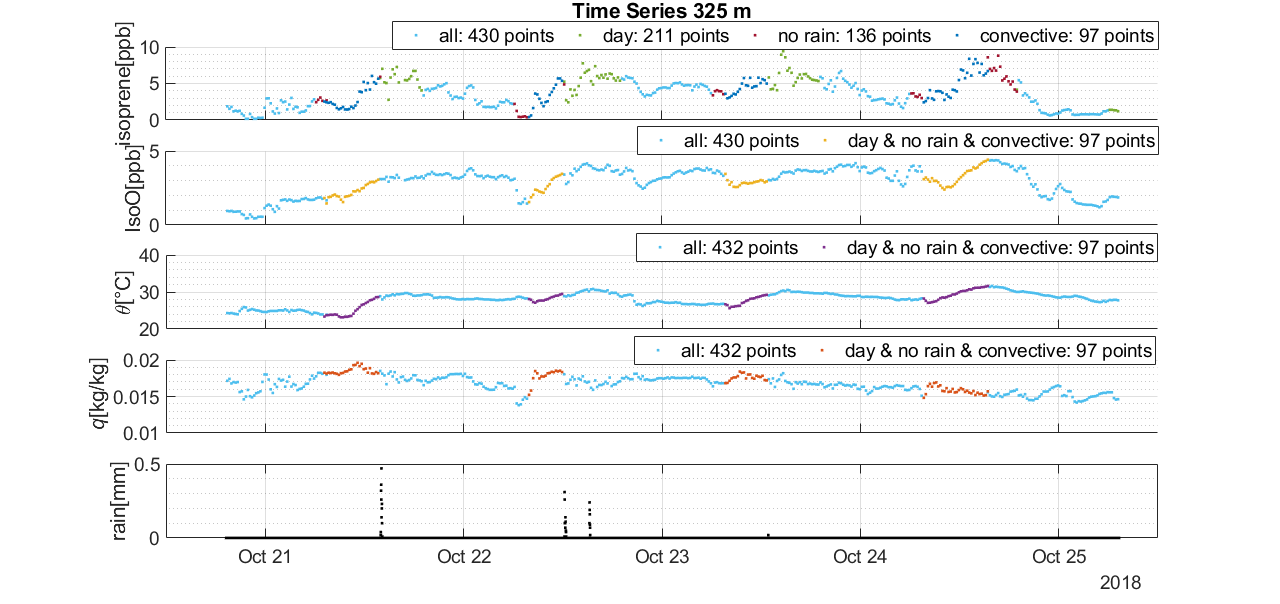


**Figure S12:** Timeseries for the measured data in the dry season 2018 of all variables at 325 m used to calculate OH concentrations. The data is averaged to 15 min resolution as applied in this study. “All” refers to the whole period with simultaneously measured isoprene, IsoO, θ, q and precipitation. “day”, “no rain” and “convective” represent the filter periods for daytime, periods with no rain (and NaN) for longer than 6 hours and convective boundary layer conditions with a negative vertical gradient of θ.


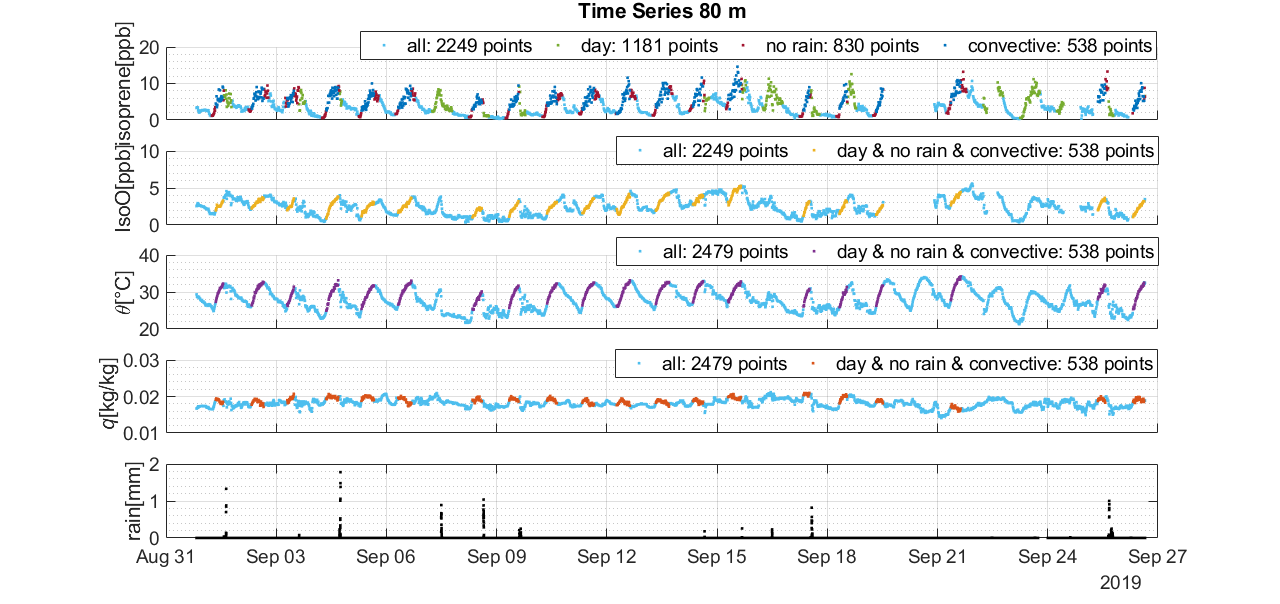


**Figure S13:** Timeseries for the measured data in the dry season 2019 of all variables at 80 m used to calculate OH concentrations. The data is averaged to 15 min resolution as applied in this study. “All” refers to the whole period with simultaneously measured isoprene, IsoO, θ, q and precipitation. “day”, “no rain” and “convective” represent the filter periods for daytime, periods with no rain (and NaN) for longer than 6 hours and convective boundary layer conditions with a negative vertical gradient of θ.


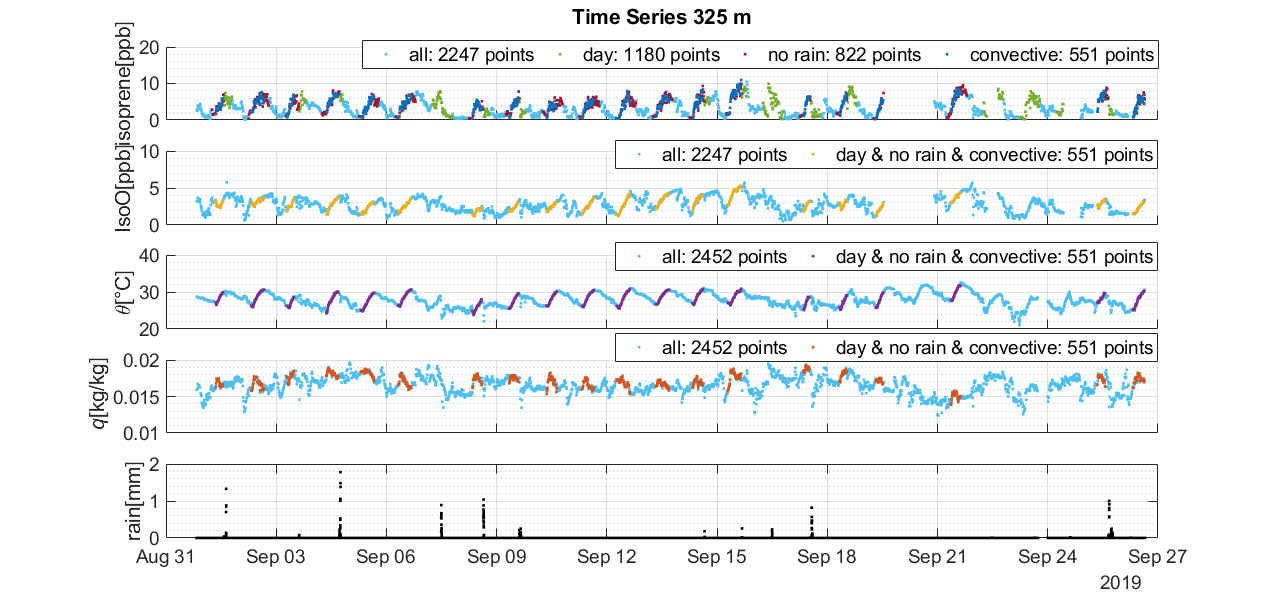


**Figure S14:** Timeseries for the measured data in the dry season 2019 of all variables at 325 m used to calculate OH concentrations. The data is averaged to 15 min resolution as applied in this study. “All” refers to the whole period with simultaneously measured isoprene, IsoO, θ, q and precipitation. “day”, “no rain” and “convective” represent the filter periods for daytime, periods with no rain (and NaN) for longer than 6 hours and convective boundary layer conditions with a negative vertical gradient of θ.


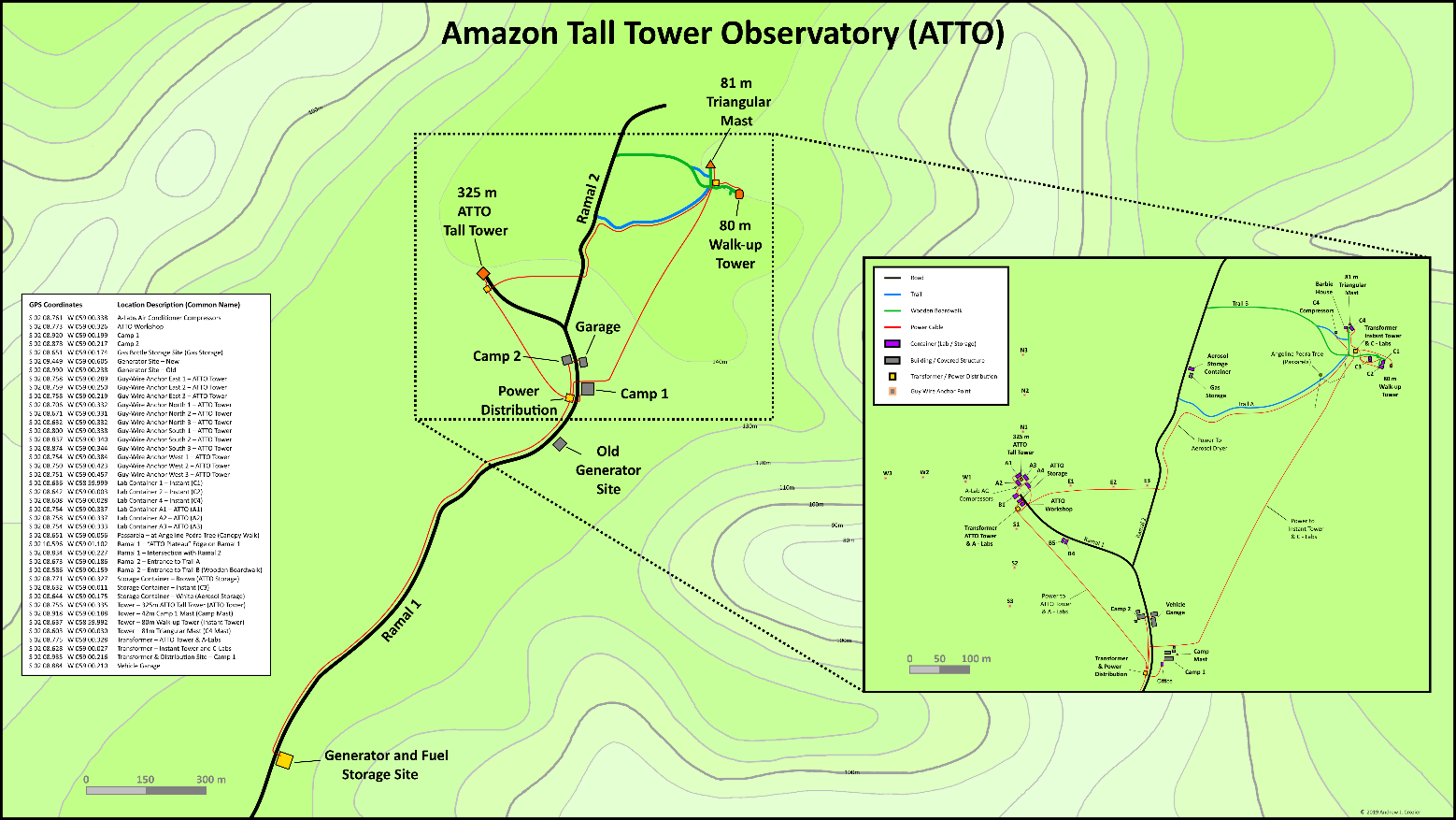


**Figure S15: A map of the ATTO site located on a plateau about 120 m above sea level.** The 80 meter high walk-up tower and the 325 meter high ATTO tower are both located on the plateau in about 1 km distance.


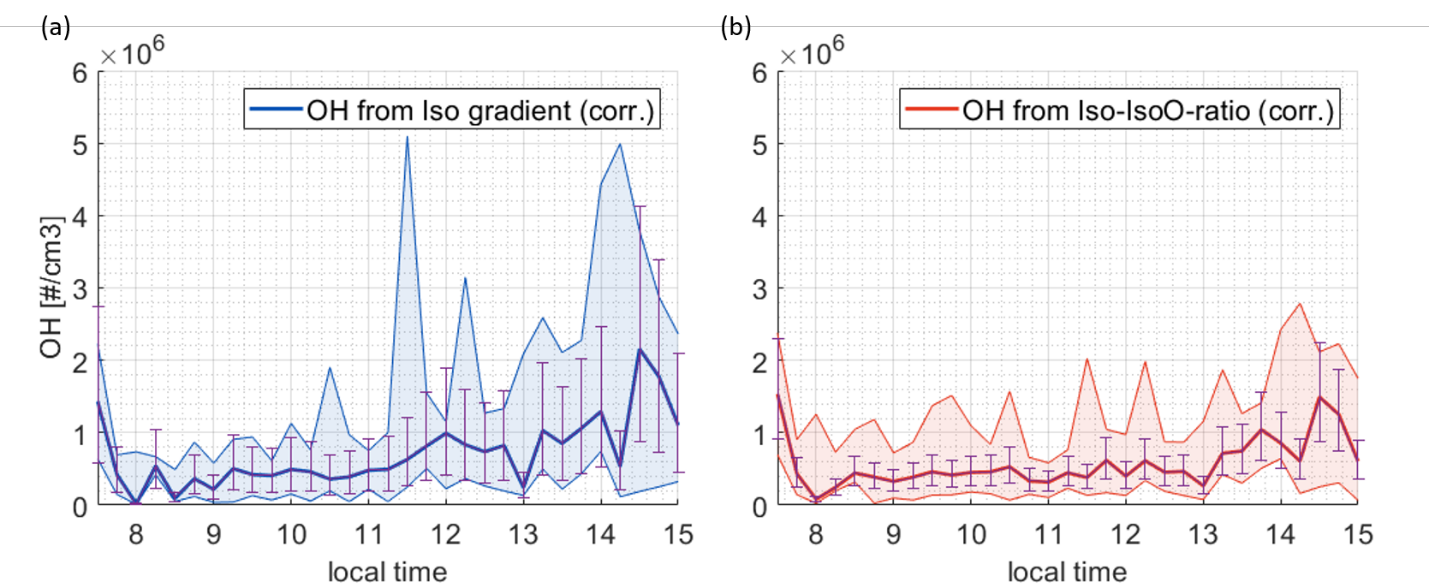


**Figure S16: OH concentrations inferred over the course of one day from ATTO observations after correcting for dynamic processes.** The shadings indicate the (0.15, 0.85) quantiles and the error bars represent the measurement uncertainty. For the mixing time, here, an uncertainty of 10 % is assumed. **a)** The daily evolution of estimated OH using the gradient method. **b)** The daily evolution of estimated OH using the ratio method.
