# Supplementary material for: Inferring the diurnal variability of OH radical concentrations over the Amazon from BVOC measurements
Source: Sci Rep. 2023 Sep 9;13:14900. doi: 10.1038/s41598-023-41748-4 (PMC10492859; doi:10.1038/s41598-023-41748-4)
Supplement: Supplementary file 2 — Supplementary Information 2. [file 41598_2023_41748_MOESM2_ESM.docx]

**## function that calculates diel cycles for one day, which are used for the DTW-function**

# applied in function dtw_height_comp()

diel_cycle_day = **function**(dataframe, d) { # the dataframe has one column representing time (“date”) and a column with the variable of interest (here: potential Temperature); d is the timestamp (POSIX format)

dd = subset(dataframe, date(dataframe$date) == d)

start_date = as.POSIXct(strptime(paste(d,'10:00:00'), format="%Y-%m-%d %H:%M:%S", tz='GMT')) # start_date is set to the time of sunrise, here 10:00 UTC

avg = timeAverage(dd, avg.time = '15 min', data.thresh = 0, statistic = 'mean', start.date=start_date, vector.ws = TRUE) # average the diurnal cycle to 15 minutes --> set time resolution here!

avg_norm = cbind(avg$date, normalize(avg[,2:dim(avg)[2]], method = "range", range = c(0, 1))); # normalize according to magnitude

avgOutput <- list("data" = avg, "normalized_data" = avg_norm)

return(avgOutput)

}

**## user defined global constraint : windowing**

# applied in function dtw_height_comp()

positiveWindow = **function**(iw, jw, query.size = n, reference.size = m){

return(jw-iw >= 0)

}

**## derive the time shift in minutes from the shift of aligned indices**

# applied in function dtw_height_comp()

indexToTT <- **function**(idx1, idx2, timeRes){

tt = c()

for ( i in 1:max(unique(idx1))){

belonging_idx = which(idx1 == i)

tt[i] = mean((idx2[belonging_idx]-i)*timeRes) # resulting time shift/transport time

}

return(tt)

}

**## function that compares 2 dataframes from different heights then calculates time shift between the curves via the difference of the two indexes, afterwards filter for convective conditions**

**## dataframe1 and 2 is typically observed at 80m and 320m respectively, time_res in minutes also specified in function diel_cycle_day()**

dtw_height_comp = function(dataframe1, dataframe2, day, time_res){

dc_height1 = diel_cycle_day(dataframe1, day) #80m

dc_height2 = diel_cycle_day(dataframe2, day) #320m

dc_length = length(dc_height2$normalized_data$poT) # poT is the potential temperature that is included in the input to the function diel_cycle_day()

alignment = dtw(dc_height1$normalized_data$poT[1:dc_length], dc_height2$normalized_data$poT[1:dc_length], step.pattern = symmetric2, window.type=positiveWindow, keep=TRUE, open.end = TRUE, open.begin = FALSE) # dynamical time warping function applied to normalized diurnal cycles of potential temperature

time_shift = indexToTT(alignment$index1, alignment$index2, 15)

max_theta = which.max(dc_height1$normalized_data$poT); # stop recording transport time when potential temperature is maximum (upper limit of filter for convective conditions)

length_dif = rep(NA, length(dc_height1$normalized_data$poT)-max_theta)

time_shift_untilMax_1 = time_shift[1:max_theta]

time_shift_untilMax = c(time_shift_untilMax_1, length_dif) # fill array with transport times, after potential temperature and therefore buoyancy is maximum fill with NA

time_axis_1 = c(format(dc_height1$data$date[1:length(dc_height1$normalized_data$poT)], format="%H:%M")) # time axis for transport times

dtwOutput <- list("transportTime" = time_shift, "transportTimeMaxTheta" = time_shift_untilMax, "timeAxis_1"=time_axis_1, "ali" = alignment, 'dc1' = dc_height1, 'dc2' = dc_height2)

return(dtwOutput)

}
